# Supplementary material for: Analysis of mental and physical disorders associated with COVID-19 in online health forums: a natural language processing study
Source: BMJ Open. 2021 Nov 5;11(11):e056601. doi: 10.1136/bmjopen-2021-056601 (PMC8573296; doi:10.1136/bmjopen-2021-056601)

Investigating mental and physical disorders associated with COVID-19 in online health forums

Supplementary Material

Methods - Search Terms and Keywords

Supplementary Table 1. Key to regular expressions

[]	One of the characters inside the bracket
+	One or more occurrences of previous character
*	Zero or more occurrences of previous character
?	Zero or one occurrence of previous character
\w	Any alphanumeric character
\s	Any whitespace character

Examples:

- 'auto[_\s-]?immune' will match all of "autoimmune", "auto-immune" and "auto immune".
- 'psycho[st]i[sc]' will match both "psychotic" and "psychosis"
- 'infarct\w*' will match "infarct", "infarcts", "infarction", "infarctions" and "infarcted"

Matching is case-insensitive. When matching post content or thread titles, keywords have to appear as separate whole words; this requirement is lifted when matching thread URLs. To reduce spurious matches, keywords up to three letters long have to appear in URLs surrounded by underscores (_) or other non-alphanumeric characters in order to be counted as a match.

Supplementary Table 2. Terms used to determine whether a thread or a post was about Covid; these were applied to both thread titles/URLs and post content

**Covid terms**	'covid', 'covid-?19', 'coronavirus', 'corona', 'sars-?cov-?2', 'sars-?2'<br>'shielding', 'pandemi\w*', 'vulnerable', 'quarantined?', 'lockdown', 'distancing', 'isolation', 'isolating'
**Discarded Covid terms**	'challenging[_\s]times?', 'difficult[_\s]times?', 'hard[_\s]times?'

Investigating mental and physical disorders associated with COVID-19 in online health forums: Supplementary Material

Supplementary Table 3. Terms used to search post content only

**Mental health symptoms**	'worried', 'worry', 'worrying', 'worries', 'anxious', 'anxiety', 'feel[_\s]low', 'feeling[_\s]low', 'depression', 'depressed', 'low[_\s]mood'
**Intensive care terms**	'itu', 'icu', 'intensive[_\s]care', 'intubation', 'intubated', 'ventilated', 'ventilator', 'c-?pap', 'ecmo', 'membrane[_\s]oxygenation'
**Physical Covid symptoms**	'chest[_\s]pain', 'smell', 'taste', 'dry[_\s]cough', 'anosmia', 'breathe?', 'breathing'

Supplementary Table 4. Term incidence of keywords used to scan thread titles/URLs for the observation period (January 1st – May 31st 2020) vs a control period (September 1st – December 31st 2019)

Keyword	Term incidence		Included/Excluded?
	Observation period (Jan 1st – May 31st 2020)	Control period (Sep 1st – December 31st 2019)	
**covid**	1395	0	Included
**covid-?19**	1205	0	Included
**coronavirus**	859	0	Included
**corona**	1140	14	Included
**sars-?cov-?2**	7	0	Included
**sars-?2**	0	0	Included
**shielding**	202	0	Included
**pandemi\w***	79	0	Included
**vulnerable**	120	3	Included
**quarantined?**	54	0	Included
**lockdown**	207	1	Included
**distancing**	82	0	Included
**isolation**	248	2	Included
**isolating**	82	0	Included
**challenging[_\s]times?**	7	0	Excluded
**difficult[_\s]times?**	13	5	Excluded
**hard[_\s]times?**	22	10	Excluded

Investigating mental and physical disorders associated with COVID-19 in online health forums: Supplementary Material

Supplementary Table 5. Keywords applied to thread titles/URLs together with Covid terms to determine threads concerning Covid comorbidities

Comorbid condition	Search terms
Heart/Stroke	'heart', 'infarct\w*', 'bypass', 'stent', 'coronary', 'ablation', 'a-?fib', 'af', 'arrhythmia', 'aortic', 'cardio', 'blockers', 'cardiom[yi]opathy', 'statins?', 'pressure', 'valve', 'pacemaker', 'stroke', 'ischa?emia', 'ischa?emic', 'anticoag', 'anticoagulants?', 'xarelto', 'apaxiban', 'rivaroxiban', 'dabigatran'
Cancer	'cancer', 'chemo', 'chemotherapy', 'tumou?rs?', 'melanoma', 'leuka?emia', 'radiation', 'radiotherapy'
Respiratory diseases	'asthma', 'asthmatic', 'copd'
Mental (health) disorders	'anxiety', 'anxious', 'depression', 'depressive', 'psycho[st]i[sc]', 'bipolar', 'schizophrenia', 'schizoaffective', 'ocd', 'ptsd'
Autoimmune diseases	'crohns', 'psoriasis', 'immunosuppress\w+', 'lupus', r'multiple[_\s]sclerosis', 'ms', 'auto[_\s-]?immune'
Diabetes	'diabet\w+', 'mellitus', 'insulin', 'humira', 'remicade', 'metformin'

Investigating mental and physical disorders associated with COVID-19 in online health forums: Supplementary Material

Investigating mental and physical disorders associated with COVID-19 in online health forums: Supplementary Material

Methods - Coding Approach

Figure 1

```
# counting users who post in threads about COVID-19 and also express a specific concern,
i.e. mention keywords from a list of search terms (e.g. mental_symptom_terms) for each
given week in time period
DEFINE count_user_concerns_week
FOR each user
    FOR each week in date_range
        IF user posted in a COVID-19 thread during week
            DEFINE sliding_window ← (previous, current and next calendar
week)
            FOR each post by user
                IF date of post in sliding_window and post content matches
regex(keywords)
                        INCREMENT count_user_concerns_week
                        BREAK   # exit innermost loop
                ENDIF
            ENDFOR
        ENDIF
    ENDFOR
ENDFOR
```

Investigating mental and physical disorders associated with COVID-19 in online health forums: Supplementary Material

Tables 1 and 2

```
DEFINE
websites                                  # list of online health forums
covid_terms                               # list of COVID-19 terms
pandemic_terms                            # list of pandemic terms
symptom_terms                             # list of physical COVID-19 symptoms
intensive_care_terms                      # list of intensive care terms
mental_symptom_terms                      # list of mental health symptoms
mental_drug_terms                         # list of drugs for mental illnesses
comorbidity_terms                         # list of comorbid conditions
date_range ← January 1st – May 31st 2020  # time period

FUNCTION regex
    INPUT list of keywords from a list of search terms
    OUTPUT compiled regex for text containing any of the keywords in the list
ENDFUNCTION
```

Table 3, Supplementary Figure 1

```
# counting posts for each given week in time period
DEFINE count_post_week
FOR each website in websites
    FOR each post on website
        FOR week in date_range
            IF date of post is in week
                INCREMENT count_post_week
            ENDIF
        ENDFOR
    ENDFOR
ENDFOR

# computing total number of posts
COMPUTE count_post_all as sum of count_post_week over date_range
```

Investigating mental and physical disorders associated with COVID-19 in online health forums: Supplementary Material

Supplementary Figure 2

```
# counting posts mentioning keywords from a list of search terms (e.g. covid_terms) for each
given week in time period
DEFINE count_relevant_post_week
FOR each website in websites
        FOR each post on website
                FOR week in date_range
                        IF date of post is in week AND post content matches regex(keywords)
                                INCREMENT count_relevant_post_week
                        ENDIF
                ENDFOR
        ENDFOR
ENDFOR
```

Note: To assess the specificity of search terms, we looked for matches before 1st January 2020, i.e. similar to the above with date_range ← < 1st January 2020

computing relative frequency of posts mentioning keywords from a list of search terms (e.g. covid_terms) for each given week in time period
COMPUTE ratio of count_relevant_post_week to count_post_week

Investigating mental and physical disorders associated with COVID-19 in online health forums: Supplementary Material

Supplementary Figure 3

```
# counting active threads about COVID-19 for each given week in time period
DEFINE count_covid_thread
FOR each website in websites
    FOR each thread on website
        IF thread title or URL matches regex(keywords from covid_terms)
            # date of first and last posts, respectively
            DEFINE active_period ← (thread.start_date, thread.end_date)
            FOR week in date_range
                IF week is in active_period    # thread is active
                    INCREMENT count_covid_thread
                ENDIF
            ENDFOR
        ENDIF
    ENDFOR
ENDFOR
```

Supplementary Figure 4

```
# counting users who post for the first time for each given week in time period
DEFINE count_first_time_user
FOR each user
    FOR week in date_range
        IF date of user's first post is in week
            INCREMENT count_first_time_user
        ENDIF
    ENDFOR
ENDFOR

# counting users whose first post is about COVID-19 for each given week in time period
DEFINE count_covid_first_time_user
FOR each user
    FOR week in date_range
        IF date of user's first post is in week AND post content matches
    regex(keywords from covid_terms)
            INCREMENT count_covid_first_time_user
        ENDIF
    ENDFOR
ENDFOR

# computing percentage of users whose first post is about COVID-19 for each given week in
time period
COMPUTE percentage of count_covid_first_time_user in count_first_time_user
```

Investigating mental and physical disorders associated with COVID-19 in online health forums: Supplementary Material

Table 4

```
# counting threads about a comorbid condition within threads about COVID-19
DEFINE count_comorbid_condition_thread
FOR each COVID-19 thread  # see code for Supplementary Figure 3
        IF thread title or URL matches regex(keyword (i.e. condition) from comorbidity_terms)
                INCREMENT count_comorbid_condition_thread
        ENDIF
ENDFOR

# computing ratio of threads about a comorbid condition to threads about COVID-19
COMPUTE ratio of count_comorbid_condition_thread to sum of count_covid_thread over date_range

# computing number of posts in threads about a comorbid condition
COMPUTE count_comorbid_condition_post as sum of number of posts in threads about a comorbid condition

# computing ratio of posts in threads about a comorbid condition to posts in threads about COVID-19
COMPUTE ratio of count_comorbid_condition_post to sum of number of posts in threads about COVID-19
```

Investigating mental and physical disorders associated with COVID-19 in online health forums: Supplementary Material

Supplementary Figure 1: Weekly number of posts on any topic in the period January 2020 to May 2020

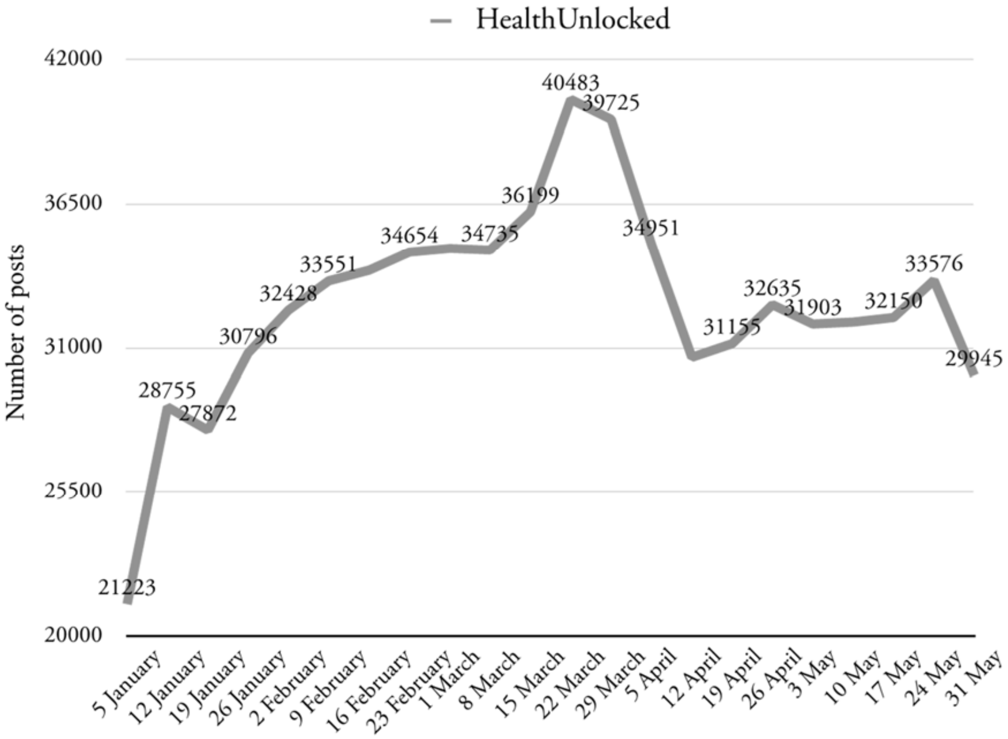

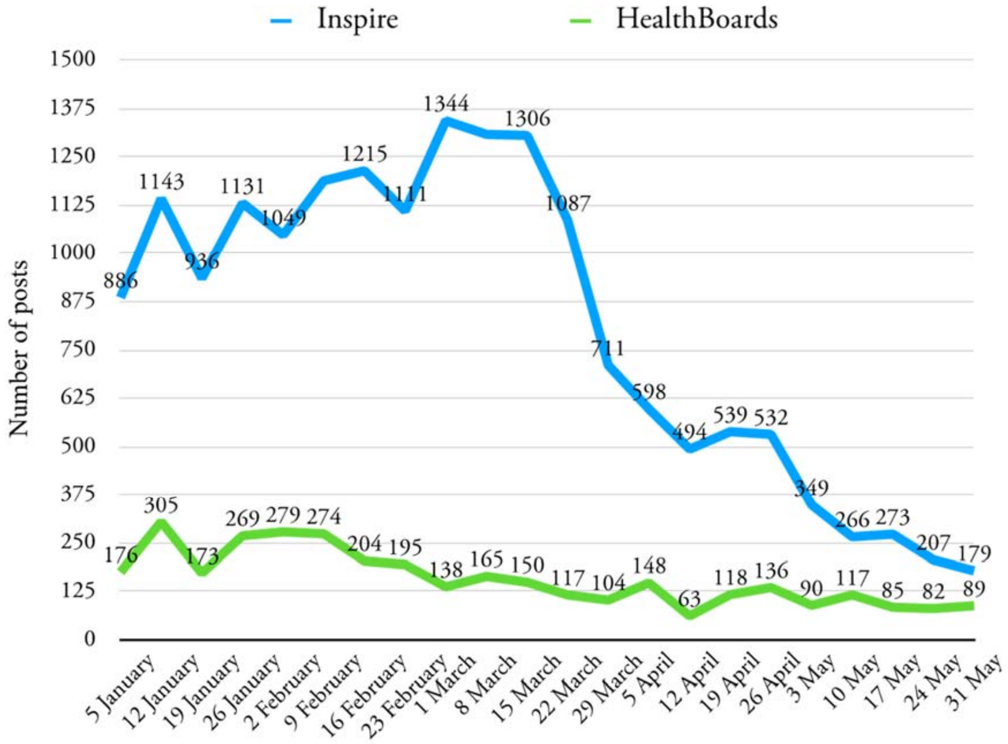

Investigating mental and physical disorders associated with COVID-19 in online health forums: Supplementary Material

Supplementary Figure 2: Percentage of COVID-19 related posts relative to all weekly posts

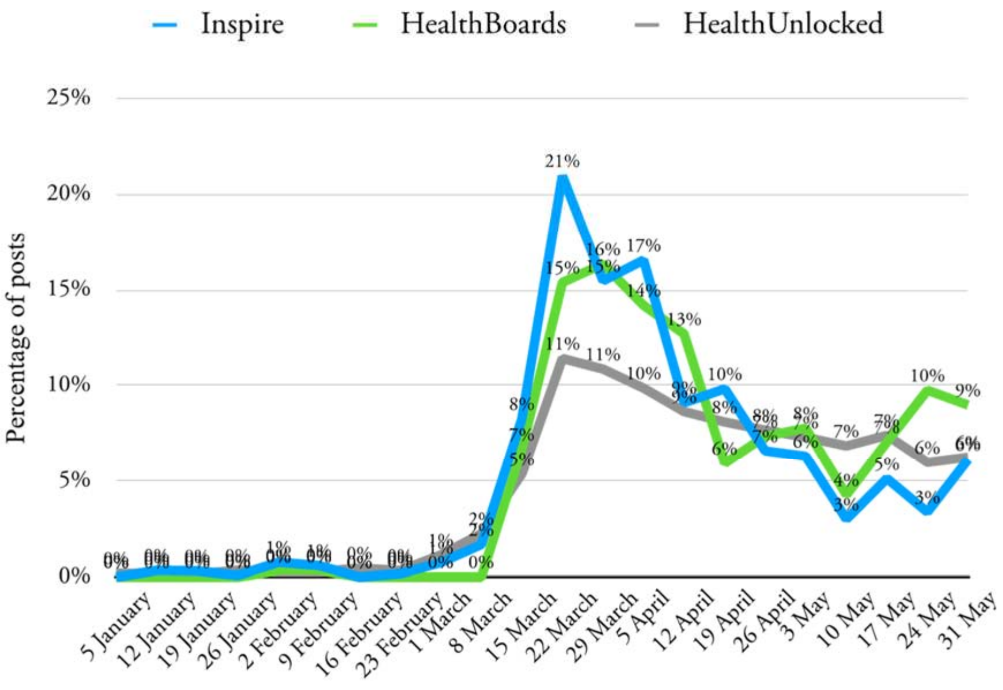

Investigating mental and physical disorders associated with COVID-19 in online health forums: Supplementary Material

Supplementary Figure 3: Maximum number of COVID-19 related threads active in each given week

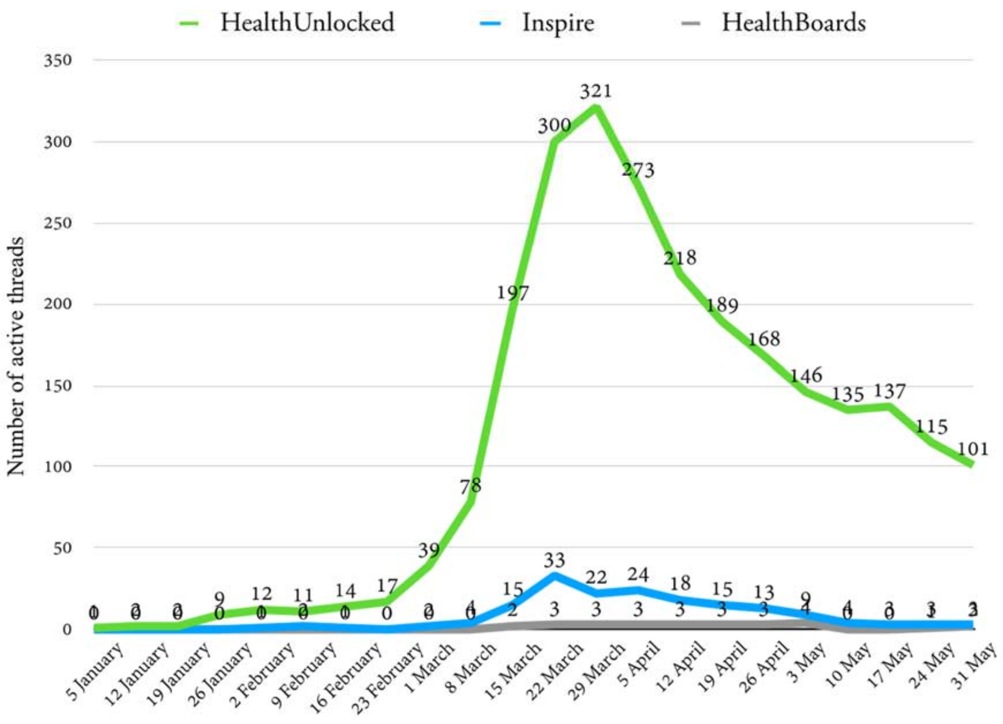

A thread is considered closed and therefore no longer active when there are no further posts from that week within the data collection period (up until 31st May 2020). Note that this does not rule out the possibility of a thread becoming reactivated past this date.

Investigating mental and physical disorders associated with COVID-19 in online health forums: Supplementary Material

Supplementary Figure 4: Proportion of users whose very first post was in a COVID-19 related thread, given weekly

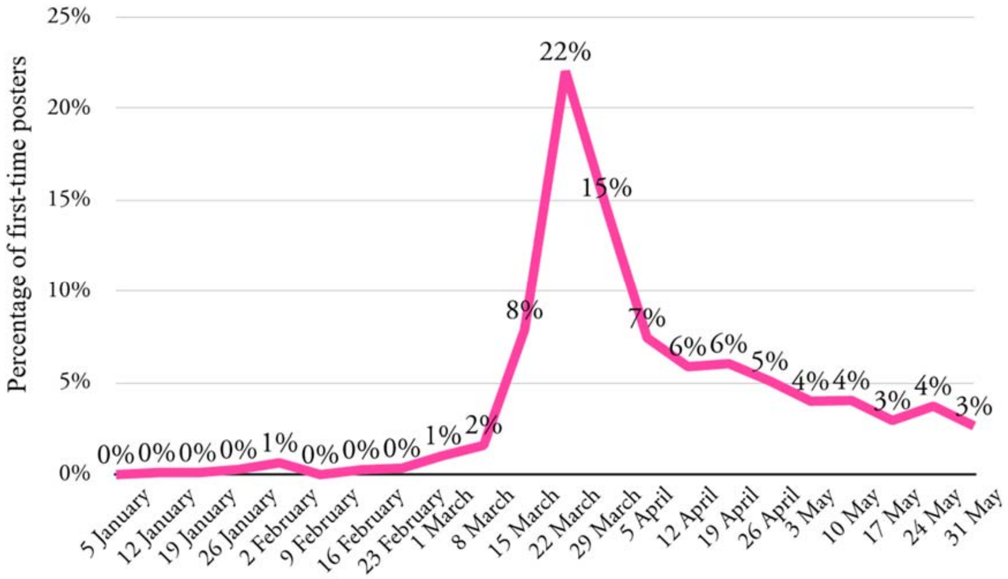

Supplement: Supplementary data [file bmjopen-2021-056601supp001.pdf]
