# Supplementary material for: Analysis of mental and physical disorders associated with COVID-19 in online health forums: a natural language processing study
Source: BMJ Open. 2021 Nov 5;11(11):e056601. doi: 10.1136/bmjopen-2021-056601 (PMC8573296; doi:10.1136/bmjopen-2021-056601)
Supplement: Author's manuscript [file bmjopen-2021-056601.draft_revisions.pdf]

BMJ Open

BMJ Open is committed to open peer review. As part of this commitment we make the peer review history of every article we publish publicly available.

When an article is published we post the peer reviewers' comments and the authors' responses online. We also post the versions of the paper that were used during peer review. These are the versions that the peer review comments apply to.

The versions of the paper that follow are the versions that were submitted during the peer review process. They are not the versions of record or the final published versions. They should not be cited or distributed as the published version of this manuscript.

BMJ Open is an open access journal and the full, final, typeset and author-corrected version of record of the manuscript is available on our site with no access controls, subscription charges or pay-per-view fees (http://bmjopen.bmj.com).

If you have any questions on BMJ Open's open peer review process please email
info.bmjopen@bmj.com

BMJ Open

Investigating mental and physical disorders associated with COVID-19 in online health forums

Journal:	*BMJ Open*
Manuscript ID	bmjopen-2021-056601
Article Type:	Original research
Date Submitted by the Author:	21-Aug-2021

Complete List of Authors:	Patel, Rashmi; King's College London Institute of Psychiatry Psychology and Neuroscience, Department of Psychosis Studies; South London and Maudsley NHS Foundation Trust<br>Smeraldi, Fabrizio; Queen Mary University of London; The Alan Turing Institute<br>Abdollahyan, Maryam; Barts Cancer Institute<br>Irving, Jessica; King's College London Institute of Psychiatry Psychology and Neuroscience, Department of Psychosis Studies<br>Bessant, Conrad; Queen Mary University of London; The Alan Turing Institute

Keywords:	COVID-19, Health informatics < BIOTECHNOLOGY & BIOINFORMATICS, Information technology < BIOTECHNOLOGY & BIOINFORMATICS

SCHOLARONE™ Manuscripts

I, the Submitting Author has the right to grant and does grant on behalf of all authors of the Work (as defined in the below author licence), an exclusive licence and/or a non-exclusive licence for contributions from authors who are: i) UK Crown employees; ii) where BMJ has agreed a CC-BY licence shall apply, and/or iii) in accordance with the terms applicable for US Federal Government officers or employees acting as part of their official duties; on a worldwide, perpetual, irrevocable, royalty-free basis to BMJ Publishing Group Ltd ("BMJ") its licensees and where the relevant Journal is co-owned by BMJ to the co-owners of the Journal, to publish the Work in this journal and any other BMJ products and to exploit all rights, as set out in our licence.

The Submitting Author accepts and understands that any supply made under these terms is made by BMJ to the Submitting Author unless you are acting as an employee on behalf of your employer or a postgraduate student of an affiliated institution which is paying any applicable article publishing charge ("APC") for Open Access articles. Where the Submitting Author wishes to make the Work available on an Open Access basis (and intends to pay the relevant APC), the terms of reuse of such Open Access shall be governed by a Creative Commons licence – details of these licences and which Creative Commons licence will apply to this Work are set out in our licence referred to above.

Other than as permitted in any relevant BMJ Author's Self Archiving Policies, I confirm this Work has not been accepted for publication elsewhere, is not being considered for publication elsewhere and does not duplicate material already published. I confirm all authors consent to publication of this Work and authorise the granting of this licence.

For peer review only

Investigating mental and physical disorders associated with COVID-19 in online health forums

Rashmi Patel[*1,2], Fabrizio Smeraldi[*3,4], Maryam Abdollahyan[5], Jessica Irving[1], and Conrad Bessant[3,4]

1. King's College London (Institute of Psychiatry, Psychology and Neuroscience), London, UK
2. South London and Maudsley NHS Foundation Trust, London, UK
3. Queen Mary University of London, UK
4. The Alan Turing Institute, London, UK
5. Barts Cancer Institute, London, UK

* Equal contribution to authorship

Correspondence to:
Rashmi Patel
E-mail: rashmi.patel@kcl.ac.uk

Manuscript word count: 3,026

Abstract word count: 220

Keywords: COVID-19, comorbidity, real world data, social media, natural language processing

For peer review only

Investigating mental and physical disorders associated with COVID-19 in online health forums:

Abstract

Objectives: Online health forums provide rich and untapped real-time data on population health. Through novel data extraction and natural language processing (NLP) techniques, we characterise the evolution of mental and physical health concerns relating to the COVID-19 pandemic among online health forum users.

Setting and design: We obtained data from 739,434 posts by 53,134 unique users of three leading online health forums: HealthBoards, Inspire and HealthUnlocked, from the period 1st January 2020 to 31st May 2020. Using NLP, we analysed the content of posts related to COVID-19.

Primary outcome measures:

(i) Proportion of forum posts containing COVID-19 keywords

(ii) Proportion of forum users making their very first post about COVID-19

(iii) Number of COVID-19 related posts containing content related to physical and mental health comorbidities

Results: Posts discussing COVID-19 and related comorbid disorders spiked in early- to mid-March around the time of global implementation of lockdowns prompting a large number of users to post on online health forums for the first time. The pandemic and corresponding public response has had a significant impact on posters' queries regarding mental health.

Conclusions: We demonstrate it is feasible to characterise the content of online health forum user posts regarding COVID-19 and measure changes over time. Social media data sources such as online health forums can be harnessed to strengthen population-level mental health surveillance.

Article Summary

Strengths and limitations of this study

1. Analysing online health forum data using NLP revealed a substantial rise in activity which correlated with the onset of the COVID-19 pandemic.
2. Real-time data sources such as online health forums are essential for monitoring fluctuating population health and tailoring responses to daily pressures.
3. It is not yet possible to establish COVID-19 status or whether concerned posters have pre-existing mental or physical health issues, are recovered, or have become unwell for the first time.
4. Online health forums are *help-seeking* forums, which introduces self-selection bias.

For peer review only

Introduction

Measures to tackle the COVID-19 pandemic have resulted in unprecedented societal restrictions worldwide. The mental health impacts of these measures and accompanying socioeconomic stressors are likely to be extensive; identifying and quantifying these impacts are now an urgent priority.[1] For example, social distancing restrictions make it harder to maintain regular contact between individuals and their friends and family as well as health and social care professionals. Furthermore, the psychological and emotional burden of the pandemic (and its consequences) may increase risk of relapse or worsen existing mental health disorders. Conversely, mental disorders can increase susceptibility to infections.[2,3]

Real-world data from online resources may be extracted using natural language processing (NLP) techniques to provide automated, population-level health surveillance. These methods can be used to rapidly ascertain discussion related to COVID-19 and associated symptoms and comorbidities. NLP has previously been used to identify medically relevant information from web pages and analyse extracted text.[4,5] Applying these techniques to real world data sources such as social media and online forums may be used to supplement active data collection from participants in prospective observational research. Recent studies have applied this approach to Twitter, Facebook and Reddit data to forecast the emergence of depression and post-traumatic stress disorder,[6] predict depression in the general population,[7] identify mothers at risk of postpartum depression,[8] and investigate suicidal ideation.[9]

While social media platforms such as Twitter, Facebook and Reddit are commonly used, other internet resources such as online health forums have so far been neglected. Online health forums are enriched for health information and receive millions of posts each year, therefore providing untapped reservoirs of healthcare data at population level.

In a recent proof-of-concept study we demonstrated that online health forums can be extracted to detect health discussion trends that correlate with real-life events.[10] Here, we use the same technology to analyse online health forum data discussing mental and physical health problems associated with the COVID-19 pandemic. We use NLP techniques to extract data from online health forum posts related to the COVID-19 pandemic, references to specific comorbid illnesses, and their direct and indirect impacts on mental or physical health.

For peer review only

Methods

Study Design and Setting

We obtained data from online health forums using NLP. Online forums are discussion websites hosted on the internet where people hold conversations in the form of posted messages. A single conversation is called a thread. Threads are chains of posts identified within a forum by a title and an individual URL. Clicking on the thread title opens the thread which contains one or more posts which may be from the same user who started the thread (i.e. the original poster) or different users who have replied within the thread. In this study, we analysed text data in thread titles and in individual posts within a thread. We analysed posts written in English only. Depending on the forum's settings, users can be anonymous or have to register with the forum to post messages, with most users opting not to use personally identifiable information to register their account. Registration may not be required for read-only access. Most forums recommend that users do not use personally identifiable information when posting. Online health forums specifically cover health topics and offer peer support for various health conditions.

We collected data from three major online health forums posted from 1st January 2020 to 31st May 2020: HealthBoards (www.healthboards.com), Inspire (www.inspire.com) and HealthUnlocked (www.healthunlocked.com). These forums were chosen on the basis that they have global user coverage, include subforums on several aspects of healthcare, have a large user base contributing to regular activity on the forum, and are feasible to extract information from using NLP.

HealthBoards was founded in California, USA in 1997 and offers patient to patient health support. Inspire, founded in 2005, is a US healthcare social network managing online support groups for patients and caregivers. HealthUnlocked is a British online health forum launched in 2011 with a similar offering to HealthBoards and Inspire. Registration and participation in all three forums are free of charge to users.

Analysis Using NLP

Extracting and matching keywords

We extracted the keywords in thread titles and post content using lemmatisation. For flexibility and efficiency, search terms in posts and thread titles were matched using regular expressions that accounted for both inflection and common spelling variants. Supplementary Table 1 provides a key to the regular expression parameters used in the study. Matching was case-insensitive and limited to whole words in the post content and thread title; when matching thread URLs, parts containing words were considered. To prevent spurious matches, words shorter than four letters (e.g. ICU) were considered valid matches only if they were delimited by non-word characters.

Definition of search terms

For peer review only

To investigate the potential impact of COVID-19 on users posting in online health forums, we classified threads and posts using keywords related to the COVID-19 pandemic and various groups of case-insensitive keywords relating to medical treatment in an intensive care unit or physical symptoms as a direct consequence of COVID-19 infection or mental health symptoms as a consequence of measures in response to the pandemic.

Search terms used to identify whether a thread or post was related to COVID-19 were 'covid', 'covid-19', 'coronavirus', 'corona', 'sars-cov-2', 'sars-2', 'shielding', 'pandemic*', 'vulnerable', 'quarantine', 'lockdown', 'distancing', 'isolation', 'isolating' where * indicates a wildcard search term.

Table 1 provides the final keywords used to search posts within COVID-19 related threads; the Python coded search terms are provided in Supplementary Tables 2 and 3. We tested the specificity of keywords by searching for matches occurring before 1st January 2020. For threads, these were matches in the title and URL, while for posts, these were matches in the entire text (see *Extracting and matching keywords* below). Term incidence and excluded keywords are provided in Supplementary Table 4.

Data pre-processing

Data obtained from different online health forums come in various formats. We standardised and normalised the data before analysing them. This included normalisation of Unicode strings and whitespace characters, standardisation of date and time, and standardisation of location through the GeoNames.org database.

Analysis of COVID-19 threads to identify changes in COVID-19 related user activity and physical and mental health associations over time

We identified the users contributing to a COVID-19 related thread in a given week. We then retrieved all the other posts made by the same authors in the previous, same and subsequent calendar weeks. We scanned such posts for physical symptom, mental health symptom or intensive care keywords as defined in Table 1, and recorded whether each of these topics was mentioned by the author during the time window. We performed this analysis to establish variations in the prevalence of concerns relating to physical symptom, mental health symptom and intensive care keywords over the course of the pandemic during 2020. Weekly counts were measured each Sunday for the previous week.

Analysis of thread titles

We inspected thread titles to identify how many mentioned a comorbidity in the title. We searched for terms related to autoimmune disorders, mental disorders or worry, cancer, cardiovascular problems or stroke, and diabetes as listed in Table 2; the Python coded search terms are provided in Supplementary Table 5.

Analysis of first-time posters in a COVID-19 related thread

We analysed the first ever post published by a user to determine the proportion of first-time posters who started out by contributing to a COVID-19 related thread. We performed this

For peer review only - http://bmjopen.bmj.com/site/about/guidelines.xhtml

analysis to determine the degree to which new users were motivated to make their first post in relation to the COVID-19 pandemic and how this varied over time during 2020.

Implementation and computation

All descriptive analyses were performed using bespoke software written in Python. An outline of the coding approach employed is included in the Supplementary Material.

Ethics and Data Sharing

The study was independently reviewed by the Queen Mary Ethics of Research Committee (QMERC) and received an exemption for ethical approval. All data were analysed on Queen Mary University of London (QMUL) IT facilities, which employs a two-layer security model as per their security policy. We consulted and adhered to internet research guidelines from the Association of Internet Researchers [11] and the British Psychological Society (BPS) [12] to inform study development.

Given licensing and privacy issues, it is not possible to publicly release the aggregate dataset generated from the three online health forums investigated. However, we welcome collaboration with other researchers and healthcare policy makers. Anyone interested in accessing the aggregate data and data analysis code should contact the guarantor (f.smeraldi@qmul.ac.uk).

Patient and public involvement

Patients and the public were not involved in the design or conduct of the study.

For peer review only

Results

Related posts and active threads

HealthUnlocked was the most frequently used forum accounting for 97% of overall posts and 97% of posts mentioning COVID-19 in the thread title or post content during the study period (Table 3).

Weekly post count for HealthUnlocked peaked in mid-March. Post count for Inspire declined sharply in the last two weeks of March. Post count for HealthBoards declined slowly across the entirety of the observation period (Supplementary Figure 1).

Across all three forums, there were a total of 3,342 threads containing a COVID-19 keyword within the thread title or URL. These contained a total of 44,894 posts during the study period (1st January 2020 to 31st May 2020). A total of 35,581 posts (whether in COVID-19 related threads or otherwise) contained a COVID-19 keyword during the study period. The proportion of posts containing COVID-19 keywords increased rapidly across all forums in early March (Supplementary Figures 2 and 3), corresponding with the World Health Organisation's declaration of COVID-19 as a pandemic on 11th March 2020. The smaller online forums (Inspire and HealthBoards) had a greater peak in percentage of total posts containing COVID-19 keywords. The total number of posts containing COVID-19 search terms declined from mid-April onwards.

For quite a long period, most posts about COVID-19 (over 90% at the beginning of the observation period, and remaining above 50% until the week ending 29th March) were written by users who had not yet posted on the topic. By the end of the observation period, the percentage of weekly posts in COVID-19 threads written by new entrants to the discussion reduced to a still quite sizeable 30%. While many of these users may have posted before on the forum about other topics, Supplementary Figure 4 presents the proportion of posters whose very first post to a forum appeared in a COVID-19 related thread. This figure peaked above 20% in the week ending 22nd March. Considering that these forums have a very broad spectrum, this is a remarkably high fraction. It includes both new joiners and users who were previously silent members of the forums, possibly for a long time (so-called "lurkers"), and who may have been spurred into a more active role by the pandemic.

Thread title analysis

Over a quarter of COVID-19 related thread titles mentioned another condition of interest (Table 4). After cancer and autoimmune diseases, mental health represented a major area of concern for online health forum users posting about COVID-19, comparable to respiratory and circulatory diseases (Table 2). Around 0.5% of thread titles mentioned two or more comorbidities.

User analysis

Posts in threads related to COVID-19 were analysed to determine the number of users contributing in each given week. For each active user, all posts in the previous, same and

For peer review only - http://bmjopen.bmj.com/site/about/guidelines.xhtml

following calendar weeks were scanned irrespective of thread for mentions of physical symptom, mental health symptom or intensive care keywords. The number of active users mentioning each of these concerns peaked in the week ending 22nd March and subsequently declined but still remained elevated above the January baseline. In particular, users discussing mental health outnumbered users mentioning the other topics (Figure 1).

For peer review only

Discussion

Using a novel technique to analyse data from online health forums, we found a marked increase in posts related to COVID-19 across the observation period of 1st January 2020 to 31st May 2020. The frequency of these posts increased rapidly in early March 2020 corresponding with the World Health Organisation's declaration of COVID-19 as a pandemic.

During this period, we found mental health symptom keywords were most frequently mentioned by authors of COVID-19 related posts (either contextually or in separate messages), followed by physical symptoms and intensive care keywords, suggesting that the pandemic and public health response to it has had a significant impact on posters' concerns regarding mental health. The marked increase in mental health symptom related posts in early March, when the WHO declared the COVID-19 pandemic, correlates with preliminary worldwide data that show increases in anxiety and depression in response to the outbreak.

The mental health impacts of COVID-19 and associated physical distancing restrictions are likely to be extensive and wide-reaching. There is a growing body of evidence supporting the neuropsychiatric effects of coronavirus infections.[13] Restrictions fuel socioeconomic stressors such as unemployment, loneliness and financial burden, which are all implicated in the development of mental ill health.[14] Increased rates of bereavement, newfound caring responsibilities and interruptions to education are likely to be particularly stressful to children and young adults.[15]

A preliminary survey of 3,545 German respondents found evidence of substantial mental health burden from travel and physical distancing restrictions, including increased levels of stress, anxiety, depressive symptoms, sleep disturbance and irritability.[16] Worsening mental health has been confirmed in samples with both pre- and post-pandemic information for direct comparison: The Avon Longitudinal Study of Parents and Children (ALSPAC) study found probable anxiety disorder doubled compared to pre-pandemic sizes (26% vs 13%) and lower wellbeing, particularly in young people, women and those with pre-existing conditions.[17] The literature on social media mining for COVID-19 mental health related trends is limited. A study analysing sentiment evolution trends of four emotions across Twitter – fear, anger, sadness, joy – has been able to identify developing shared distress, and topics of interest relating to those emotions.[18]

Our findings also suggest that mental and physical health concerns documented in online forum posts have levelled off following their peak in March 2020. The number of users active in COVID-19 threads who also wrote posts concerning mental health symptoms reduced from their peak in March of 1,355 (per week) to 253 by the end of the observation period (compared to a mean number of 30 per week in January), suggesting that as time went on most users had begun to adjust to the consequences of the pandemic. Other NLP studies have also identified a similar trend. An analysis of 10 million Google searches within the United States found large shifts in mental health symptom searches linked to stay-at-home orders in the United States across the week commencing 16th March 2020.[19] Searches for topics related to anxiety,

For peer review only - http://bmjopen.bmj.com/site/about/guidelines.xhtml

negative thoughts about oneself and the future, insomnia and suicidal ideation dramatically increased prior to stay-at-home orders, levelling off upon the announcement of stay-at-home orders. These patterns were relatively unique to searches for mental health related information and not physical conditions.

Over the entire period, on average 4% of first-time posters (over 20% in the peak period) made their very first contribution to the forum in a COVID-19 related thread. Furthermore, 77% of COVID-19 threads were started by users who had never posted about the topic before, and chose to start out by creating their own thread. A certain degree of motivation is required to take someone to the point of making that first post on a forum, and also for starting a thread; our finding suggests that the pandemic is driving users to engage more actively in community forum services in times of uncertainty.

Strengths and weaknesses

Online health forums are an important source of real-world, real-time, population-level data on people living through the COVID-19 pandemic. Online health forums also afford users anonymity to discuss aspects of their experience they might otherwise have been embarrassed or fearful to disclose in identifiable forms of social media. We have demonstrated that it is possible to automate information extraction from these posts using natural language processing, providing access to a rich reservoir of previously untapped real-world data from health-specific online resources.

Our approach was able to automatically extract data from a large sample of over 53,000 unique users at a fraction of the cost of previous approaches that have relied on social media individual participant recruitment and manual review of posts generating sample sizes in the low hundreds.[7] Some studies screened users on Twitter via depression symptom questionnaires and used their tweets to train depression onset classifiers.[6,20] Analogous approaches have been used with Facebook data.[8]

Our study has some limitations. At present it is difficult to establish whether concerned posters have pre-existing mental or physical health issues, have experienced confirmed COVID-19 illness themselves, are recovered, or have become unwell for the first time. Online health forums are help-seeking communities; this introduces self-selection bias in which individuals from disadvantaged backgrounds who do not have IT equipment/network connection to access online resources are under-represented and our results are therefore not generalisable to the entire population. Furthermore, as these forums have worldwide coverage we cannot isolate trends to one geographic region. However, future work could utilise the location data (see *Data pre-processing* in Methods) to explore this avenue.

Conclusions and future research

Publicly accessible sources of real-world data, such as online health forums analysed in this study, can strengthen population-level physical and mental health surveillance and provide a rapid and inexpensive means to inform public healthcare policy. We found that the majority of posts in online forum data related to COVID-19 concerned features related to mental health

For peer review only

and that the peak in frequency of posts corresponded with the early phase of the pandemic, indicating the significant impact of COVID-19 on the mental health of susceptible populations.

As the pandemic evolves, further research using online forum data could improve our understanding of the long-term consequences of COVID-19 infection [21] and the longer-term socioeconomic consequences of travel and physical distancing restrictions that have been employed in many countries to manage viral transmission.[22,23] Analysis of real-world data, including social media and online health forums, could provide a useful insight into attitudes and perceptions towards novel therapeutics. This will be crucial to maximising uptake of effective preventative approaches such as mask-wearing, physical distancing, hygiene measures and potential vaccines.

For peer review only

Competing interests: All authors have completed the ICMJE uniform disclosure form at www.icmje.org/coi_disclosure.pdf and declare: RP has received funds from Janssen, Induction Healthcare and Holmusk outside the current study. The other authors declare no competing interests.

Contributorship statement: The study was conceived by RP and FS. Data extraction and statistical analysis were performed by FS, MA and CB. Reporting of findings were carried out by RP and JI. All authors (RP, FS, MA, JI and CB) contributed to study design, manuscript preparation and approved the final version. FS is guarantor.

Ethics approval: Data for this study were drawn from publicly available online health forums and extracted in aggregate form for secondary data analysis rather than at individual user level. The study was independently reviewed by the Queen Mary Ethics of Research Committee (QMERC) and received an exemption for ethical approval. The data were analysed using the computing infrastructure based at Queen Mary University of London (QMUL) which employs a two-layer security model to maintain data privacy. QMUL is registered as a data controller with the Information Commissioner's Office (ICO; registration number: Z5507327), which covers all research activities undertaken at the university.

Source of funding: RP has received support from a Medical Research Council (MRC) Health Data Research UK Fellowship (MR/S003118/1) and a Starter Grant for Clinical Lecturers (SGL015/1020) supported by the Academy of Medical Sciences, The Wellcome Trust, MRC, British Heart Foundation, Arthritis Research UK, the Royal College of Physicians and Diabetes UK. FS and CB were funded by an Alan Turing Institute (ATI) Fellowship and by an EPSRC COVID-19 Rapid Response Impact Acceleration Fund. Computational resources were funded by a Microsoft Azure Sponsorship through the ATI.

Role of funder: The views expressed are those of the authors and not necessarily those of the funders. The funders had no role in the design and conduct of the study; collection, management, analysis, and interpretation of the data; preparation, review, or approval of the manuscript; and decision to submit the manuscript for publication.

Data sharing: Given licensing and privacy issues, it is not possible to release the dataset generated from the online health forums investigated in this study. However, we welcome collaboration with other researchers and healthcare policy makers. Anyone interested in accessing the aggregate data and data analysis code should contact the guarantor (f.smeraldi@qmul.ac.uk).

For peer review only

References

1 Holmes EA, O'Connor RC, Perry VH, *et al.* Multidisciplinary research priorities for the COVID-19 pandemic: a call for action for mental health science. The Lancet Psychiatry. 2020;**7**:547–60. doi:10.1016/S2215-0366(20)30168-1

2 Yao H, Chen J-H, Xu Y-F. Patients with mental health disorders in the COVID-19 epidemic. 2020. doi:10.1016/S2215-0366(20)30090-0

3 Seminog OO, Goldacre MJ. Risk of pneumonia and pneumococcal disease in people with severe mental illness: English record linkage studies. *Thorax* Published Online First: 2013. doi:10.1136/thoraxjnl-2012-202480

4 Landers RN, Brusso RC, Cavanaugh KJ, *et al.* A primer on theory-driven web scraping: Automatic extraction of big data from the internet for use in psychological research. *Psychol Methods* 2016;**21**:475–92. doi:10.1037/met0000081

5 Cambria E, White B. Jumping NLP curves: A review of natural language processing research. IEEE Comput. Intell. Mag. 2014;**9**:48–57. doi:10.1109/MCI.2014.2307227

6 Reece AG, Reagan AJ, Lix KLM, *et al.* Forecasting the onset and course of mental illness with Twitter data. *Sci Rep* 2017;**7**:1–11. doi:10.1038/s41598-017-12961-9

7 De Choudhury M, Gamon M, Counts S, *et al.* Predicting Depression via Social Media. 2013.

8 De Choudhury M, Counts S, Horvitz E. Predicting postpartum changes in emotion and behavior via social media. In: *Conference on Human Factors in Computing Systems - Proceedings*. New York, New York, USA: : ACM Press 2013. 3267–76. doi:10.1145/2470654.2466447

9 De Choudhury M, Kiciman E, Dredze M, *et al.* Discovering shifts to suicidal ideation from mental health content in social media. In: *Conference on Human Factors in Computing Systems - Proceedings*. Association for Computing Machinery 2016. 2098–110. doi:10.1145/2858036.2858207

10 Abdollahyan M, Smeraldi F, Patel R, *et al.* Investigating Comorbidity of Mental and Physical Disorders in On-line Health Forums. Published Online First: 2020. doi:10.1145/3378184.3378195

11 Franzke AS, Bechmann A, Zimmer M, *et al.* Internet Research: Ethical Guidelines 3.0. 2020.

12 British Psychological Society. British Psychological Society Ethics Guidelines for Internet-mediated Research. *Br Psychol Soc* Published Online First: 2017. doi:INF206/1.2013

13 Rogers JP, Chesney E, Oliver D, *et al.* Psychiatric and neuropsychiatric presentations associated with severe coronavirus infections: a systematic review and meta-analysis with comparison to the COVID-19 pandemic. *The Lancet Psychiatry* 2020;**7**:611–27. doi:10.1016/S2215-0366(20)30203-0

14 Kawohl W, Nordt C. COVID-19, unemployment, and suicide. The Lancet Psychiatry. 2020;**7**:389–90. doi:10.1016/S2215-0366(20)30141-3

15 McManus S, Bebbington P, Jenkins R, *et al.* Mental health and wellbeing in England. 2016.

16 Jung S, Kneer J, Tillmann HC, *et al.* The German COVID-19 Survey on Mental Health: Primary Results. doi:10.1101/2020.05.06.20090340

17 F Kwong AS, Pearson M, Adams J, *et al.* Mental health during the COVID-19 pandemic in two longitudinal UK population cohorts 1. doi:10.1101/2020.06.16.20133116

18 Lwin MO, Lu J, Sheldenkar A, *et al.* Global Sentiments Surrounding the COVID-19 Pandemic on Twitter: Analysis of Twitter Trends. doi:10.2196/19447

19 Jacobson NC, Lekkas D, Price G, *et al.* Flattening the Mental Health Curve: COVID-19 Stay-at-Home Orders Are Associated With Alterations in Mental Health Search Behavior in the United States. *JMIR Ment Heal* Published Online First: 2020. doi:10.2196/19347

20 Tsugawa S, Kikuchi Y, Kishino F, *et al.* Recognizing depression from twitter activity. In: *Conference on Human Factors in Computing Systems - Proceedings*. New York, New York, USA: : Association for Computing Machinery 2015. 3187–96. doi:10.1145/2702123.2702280

21 Mahase E. Covid-19: What do we know about 'long covid'? BMJ. 2020;**370**. doi:10.1136/bmj.m2815

22 Rossi R, Socci V, Talevi D, *et al.* COVID-19 Pandemic and Lockdown Measures Impact on

For peer review only

Mental Health Among the General Population in Italy. *Front Psychiatry* 2020;**11**. doi:10.3389/fpsyt.2020.00790
23 Veer IM, Riepenhausen A, Zerban M, *et al.* Psycho-social factors associated with mental resilience in the Corona lockdown. doi:10.31234/OSF.IO/4Z62T

For peer review only

Investigating mental and physical disorders associated with COVID-19 in online health forums:

Figure 1 legend:

Number of users making posts in threads related to COVID-19 which included physical symptoms, mental health symptoms or intensive care keywords

For peer review only

Investigating mental and physical disorders associated with COVID-19 in online health forums:

Table 1: Physical health, mental health and intensive care keywords used to search threads and posts

**Physical COVID-19 symptoms**	chest pain, smell, taste, dry cough, anosmia, breath*
**Mental health symptoms**	worried, worry, worrying, worries, anxious, anxiety, feel(s) low, feeling low, depression, depressed, low mood
**Intensive care terms**	itu, icu, intensive care, intubation, intubated, ventilated, ventilator, c-pap, ecmo, membrane oxygenation

Table 2. Keywords applied to threads containing COVID-19 search terms to investigate physical and mental health diagnoses potentially associated with the COVID-19 pandemic

**Comorbid condition**	**Search terms**
Cardiovascular/stroke	heart, infarct*, bypass, stent, coronary, ablation, atrial fibrillation, arrhythmia, aortic, cardio*, blockers, cardiomyopathy, statin(s), pressure, valve, pacemaker, stroke, ischaemia, ischaemic, anticoag*, anticoagulants, xarelto, apaxiban, rivaroxiban, dabigatran
Cancer	cancer, chemo, chemotherapy, tumours, melanoma, leukaemia, radiation, radiotherapy
Respiratory diseases	asthma, asthmatic, copd
Mental disorders	anxiety, anxious, depression, depressive, psychosis, psychotic, bipolar, schizophrenia, schizoaffective, ocd, ptsd
Autoimmune diseases	crohns, psoriasis, immunosuppress(ant), lupus, multiple sclerosis, ms, auto-immune
Diabetes	diabetes, mellitus, insulin, humira, remicade, metformin

* denotes a wildcard search

For peer review only

Table 3: Number of users and posts retrieved from selected online health forums from 1st January 2020 to 31st May 2020

Website	Number of users[a]	Number of posts	Number (%) of posts mentioning COVID-19
HealthUnlocked *(www.healthunlocked.com)*	47,999	718,103	34,657 (4.8%)
HealthBoards *(www.healthboards.com)*	601	3,477	127 (3.7%)
Inspire *(www.inspire.com)*	4,534	17,854	797 (4.5%)
**Total**	**53,134**	**739,434**	**35,581 (4.8%)**

[a] A user is defined as anyone who posted within the data collection period

Table 4: Number and proportion of COVID-19 related threads and posts contained within COVID-19 related threads that mention a comorbidity

Comorbidity	Number of threads, n (%)[a]	Number of posts contained within threads, n (%)[b]
No condition of interest	2,340 (70.0%)	34,201 (76.2%)
Autoimmune disorders	254 (7.6%)	2,739 (6.1%)
Cancer	223 (6.7%)	2,114 (4.7%)
Respiratory	150 (4.5%)	1,905 (4.2%)
Cardiovascular / Stroke	133 (4.0%)	1,583 (3.5%)
Diabetes	45 (1.3%)	295 (0.7%)
Mental disorders	215 (6.4%)	2,243 (5.0%)

[a] Percentage is calculated as number of mentions of comorbidities/ total threads (N = 3,342).
[b] Percentage is calculated as number of posts / total posts (N = 44,894)
Note: Percentages do not add up to 100% because some threads contained mentions of more than one comorbidity

For peer review only

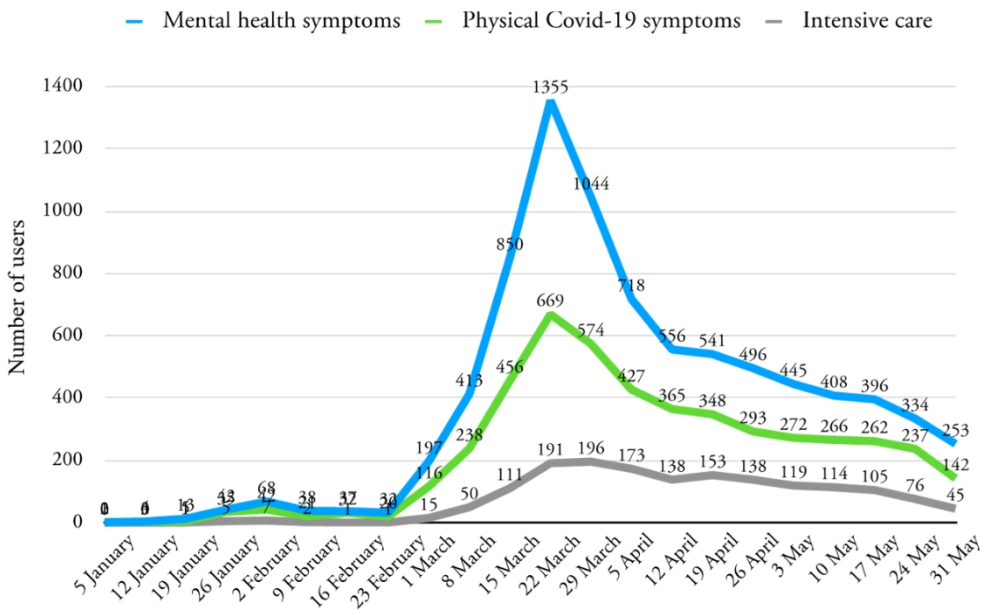

Figure 1: Number of users making posts in threads related to COVID-19 which included physical symptoms, mental health symptoms or intensive care keywords

108x67mm (300 x 300 DPI)

Investigating mental and physical disorders associated with COVID-19 in online health forums

Supplementary Material

Methods - Search Terms and Keywords

Supplementary Table 1. Key to regular expressions

[]	One of the characters inside the bracket
+	One or more occurrences of previous character
*	Zero or more occurrences of previous character
?	Zero or one occurrence of previous character
\w	Any alphanumeric character
\s	Any whitespace character

Examples:

- 'auto[_\s-]?immune' will match all of "autoimmune", "auto-immune" and "auto immune".
- 'psycho[st]i[sc]' will match both "psychotic" and "psychosis"
- 'infarct\w*' will match "infarct", "infarcts", "infarction", "infarctions" and "infarcted"

Matching is case-insensitive. When matching post content or thread titles, keywords have to appear as separate whole words; this requirement is lifted when matching thread URLs. To reduce spurious matches, keywords up to three letters long have to appear in URLs surrounded by underscores (_) or other non-alphanumeric characters in order to be counted as a match.

Supplementary Table 2. Terms used to determine whether a thread or a post was about Covid; these were applied to both thread titles/URLs and post content

**Covid terms**	'covid', 'covid-?19', 'coronavirus', 'corona', 'sars-?cov-?2', 'sars-?2'<br>'shielding', 'pandemi\w*', 'vulnerable', 'quarantined?', 'lockdown', 'distancing', 'isolation', 'isolating'
**Discarded Covid terms**	'challenging[_\s]times?', 'difficult[_\s]times?', 'hard[_\s]times?'

For peer review only

Investigating mental and physical disorders associated with COVID-19 in online health forums: Supplementary Material

Supplementary Table 3. Terms used to search post content only

**Mental health symptoms**	'worried', 'worry', 'worrying', 'worries', 'anxious', 'anxiety', 'feel[_\s]low', 'feeling[_\s]low', 'depression', 'depressed', 'low[_\s]mood'
**Intensive care terms**	'itu', 'icu', 'intensive[_\s]care', 'intubation', 'intubated', 'ventilated', 'ventilator', 'c-?pap', 'ecmo', 'membrane[_\s]oxygenation'
**Physical Covid symptoms**	'chest[_\s]pain', 'smell', 'taste', 'dry[_\s]cough', 'anosmia', 'breathe?', 'breathing'

Supplementary Table 4. Term incidence of keywords used to scan thread titles/URLs for the observation period (January 1st – May 31st 2020) vs a control period (September 1st – December 31st 2019)

**Keyword**	**Term incidence**		**Included/Excluded?**
	**Observation period (Jan 1st – May 31st 2020)**	**Control period (Sep 1st – December 31st 2019)**	
**covid**	1395	0	Included
**covid-?19**	1205	0	Included
**coronavirus**	859	0	Included
**corona**	1140	14	Included
**sars-?cov-?2**	7	0	Included
**sars-?2**	0	0	Included
**shielding**	202	0	Included
**pandemi\w***	79	0	Included
**vulnerable**	120	3	Included
**quarantined?**	54	0	Included
**lockdown**	207	1	Included
**distancing**	82	0	Included
**isolation**	248	2	Included
**isolating**	82	0	Included
**challenging[_\s]times?**	7	0	Excluded
**difficult[_\s]times?**	13	5	Excluded
**hard[_\s]times?**	22	10	Excluded

For peer review only

Investigating mental and physical disorders associated with COVID-19 in online health forums: Supplementary Material

Supplementary Table 5. Keywords applied to thread titles/URLs together with Covid terms to determine threads concerning Covid comorbidities

Comorbid condition	Search terms
Heart/Stroke	'heart', 'infarct\w*', 'bypass', 'stent', 'coronary', 'ablation', 'a-?fib', 'af', 'arrhythmia', 'aortic', 'cardio', 'blockers', 'cardiom[yi]opathy', 'statins?', 'pressure', 'valve', 'pacemaker', 'stroke', 'ischa?emia', 'ischa?emic', 'anticoag', 'anticoagulants?', 'xarelto', 'apaxiban', 'rivaroxiban', 'dabigatran'
Cancer	'cancer', 'chemo', 'chemotherapy', 'tumou?rs?', 'melanoma', 'leuka?emia', 'radiation', 'radiotherapy'
Respiratory diseases	'asthma', 'asthmatic', 'copd'
Mental (health) disorders	'anxiety', 'anxious', 'depression', 'depressive', 'psycho[st]i[sc]', 'bipolar', 'schizophrenia', 'schizoaffective', 'ocd', 'ptsd'
Autoimmune diseases	'crohns', 'psoriasis', 'immunosuppress\w+', 'lupus', r'multiple[_\s]sclerosis', 'ms', 'auto[_\s-]?immune'
Diabetes	'diabet\w+', 'mellitus', 'insulin', 'humira', 'remicade', 'metformin'

For peer review only

Investigating mental and physical disorders associated with COVID-19 in online health forums: Supplementary Material

For peer review only

Investigating mental and physical disorders associated with COVID-19 in online health forums: Supplementary Material

Methods - Coding Approach

Figure 1

```
# counting users who post in threads about COVID-19 and also express a specific concern,
i.e. mention keywords from a list of search terms (e.g. mental_symptom_terms) for each
given week in time period
DEFINE count_user_concerns_week
FOR each user
        FOR each week in date_range
                IF user posted in a COVID-19 thread during week
                        DEFINE sliding_window ← (previous, current and next calendar
week)
                        FOR each post by user
                                IF date of post in sliding_window and post content matches
regex(keywords)
                                        INCREMENT count_user_concerns_week
                                        BREAK   # exit innermost loop
                                ENDIF
                        ENDFOR
                ENDIF
        ENDFOR
ENDFOR
```

For peer review only

Investigating mental and physical disorders associated with COVID-19 in online health forums: Supplementary Material

Tables 1 and 2

```
DEFINE
websites                                # list of online health forums
covid_terms                             # list of COVID-19 terms
pandemic_terms                          # list of pandemic terms
symptom_terms                           # list of physical COVID-19 symptoms
intensive_care_terms                    # list of intensive care terms
mental_symptom_terms                    # list of mental health symptoms
mental_drug_terms                       # list of drugs for mental illnesses
comorbidity_terms                       # list of comorbid conditions
date_range ← January 1st – May 31st 2020   # time period

FUNCTION regex
    INPUT list of keywords from a list of search terms
    OUTPUT compiled regex for text containing any of the keywords in the list
ENDFUNCTION
```

Table 3, Supplementary Figure 1

```
# counting posts for each given week in time period
DEFINE count_post_week
FOR each website in websites
    FOR each post on website
        FOR week in date_range
            IF date of post is in week
                INCREMENT count_post_week
            ENDIF
        ENDFOR
    ENDFOR
ENDFOR

# computing total number of posts
COMPUTE count_post_all as sum of count_post_week over date_range
```

For peer review only

Investigating mental and physical disorders associated with COVID-19 in online health forums: Supplementary Material

Supplementary Figure 2

```
# counting posts mentioning keywords from a list of search terms (e.g. covid_terms) for each given week in time period
DEFINE count_relevant_post_week
FOR each website in websites
        FOR each post on website
                FOR week in date_range
                        IF date of post is in week AND post content matches regex(keywords)
                                INCREMENT count_relevant_post_week
                        ENDIF
                ENDFOR
        ENDFOR
ENDFOR

# Note: To assess the specificity of search terms, we looked for matches before 1st January 2020, i.e. similar to the above with date_range ← < 1st January 2020

# computing relative frequency of posts mentioning keywords from a list of search terms (e.g. covid_terms) for each given week in time period
COMPUTE ratio of count_relevant_post_week to count_post_week
```

For peer review only

Investigating mental and physical disorders associated with COVID-19 in online health forums: Supplementary Material

Supplementary Figure 3

```
# counting active threads about COVID-19 for each given week in time period
DEFINE count_covid_thread
FOR each website in websites
        FOR each thread on website
                IF thread title or URL matches regex(keywords from covid_terms)
                        # date of first and last posts, respectively
                        DEFINE active_period ← (thread.start_date, thread.end_date)
                        FOR week in date_range
                                IF week is in active_period    # thread is active
                                        INCREMENT count_covid_thread
                                ENDIF
                        ENDFOR
                ENDIF
        ENDFOR
ENDFOR
```

Supplementary Figure 4

```
# counting users who post for the first time for each given week in time period
DEFINE count_first_time_user
FOR each user
        FOR week in date_range
                IF date of user's first post is in week
                        INCREMENT count_first_time_user
                ENDIF
        ENDFOR
ENDFOR

# counting users whose first post is about COVID-19 for each given week in time period
DEFINE count_covid_first_time_user
FOR each user
        FOR week in date_range
                IF date of user's first post is in week AND post content matches
        regex(keywords from covid_terms)
                        INCREMENT count_covid_first_time_user
                ENDIF
        ENDFOR
ENDFOR

# computing percentage of users whose first post is about COVID-19 for each given week in
time period
COMPUTE percentage of count_covid_first_time_user in count_first_time_user
```

For peer review only

Investigating mental and physical disorders associated with COVID-19 in online health forums: Supplementary Material

Table 4

```
# counting threads about a comorbid condition within threads about COVID-19
DEFINE count_comorbid_condition_thread
FOR each COVID-19 thread  # see code for Supplementary Figure 3
	IF thread title or URL matches regex(keyword (i.e. condition) from comorbidity_terms)
		INCREMENT count_comorbid_condition_thread
	ENDIF
ENDFOR

# computing ratio of threads about a comorbid condition to threads about COVID-19
COMPUTE ratio of count_comorbid_condition_thread to sum of count_covid_thread over date_range

# computing number of posts in threads about a comorbid condition
COMPUTE count_comorbid_condition_post as sum of number of posts in threads about a comorbid condition

# computing ratio of posts in threads about a comorbid condition to posts in threads about COVID-19
COMPUTE ratio of count_comorbid_condition_post to sum of number of posts in threads about COVID-19
```

For peer review only

Investigating mental and physical disorders associated with COVID-19 in online health forums: Supplementary Material

Supplementary Figure 1: Weekly number of posts on any topic in the period January 2020 to May 2020

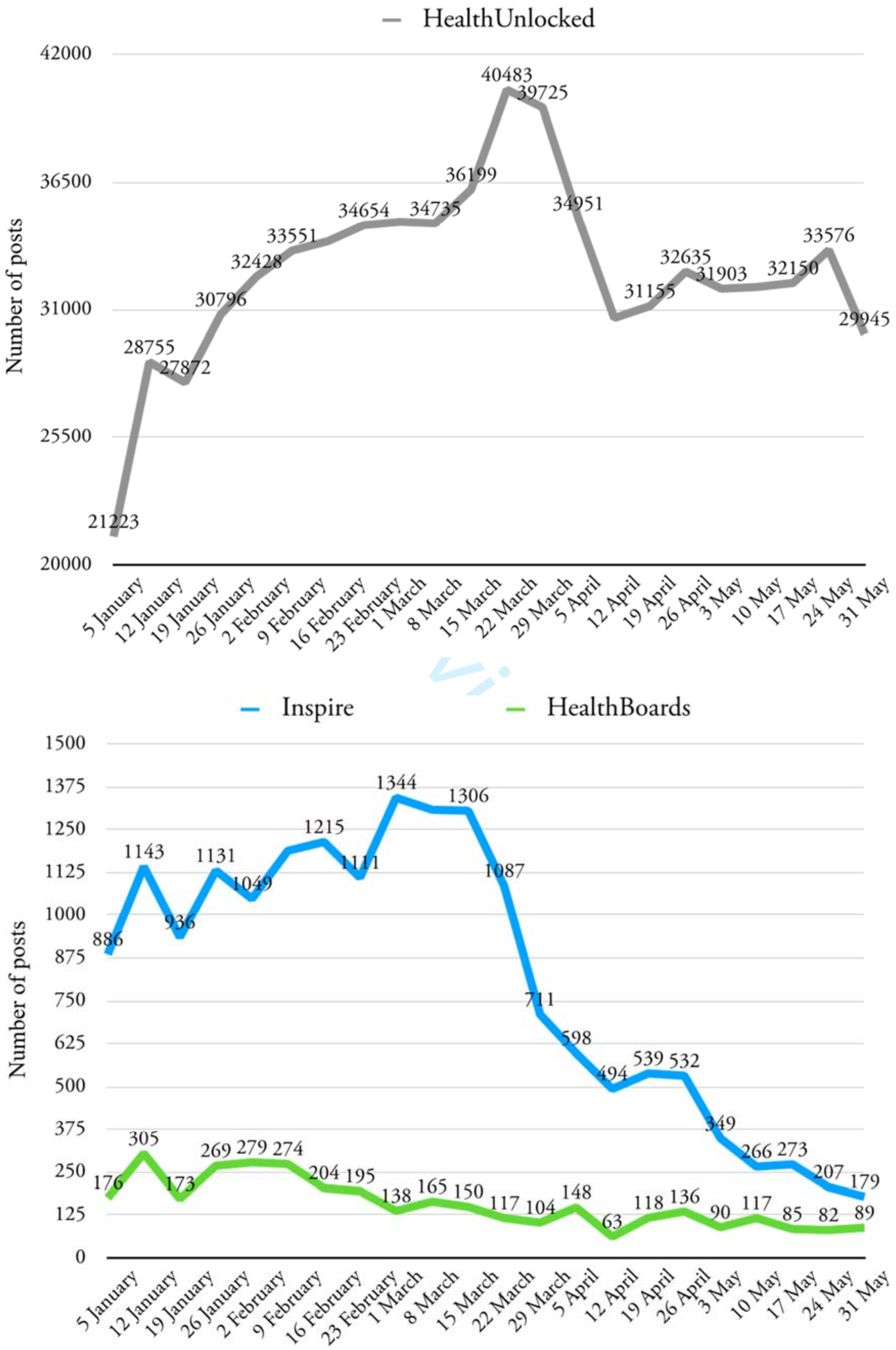

Investigating mental and physical disorders associated with COVID-19 in online health forums: Supplementary Material

Supplementary Figure 2: Percentage of COVID-19 related posts relative to all weekly posts

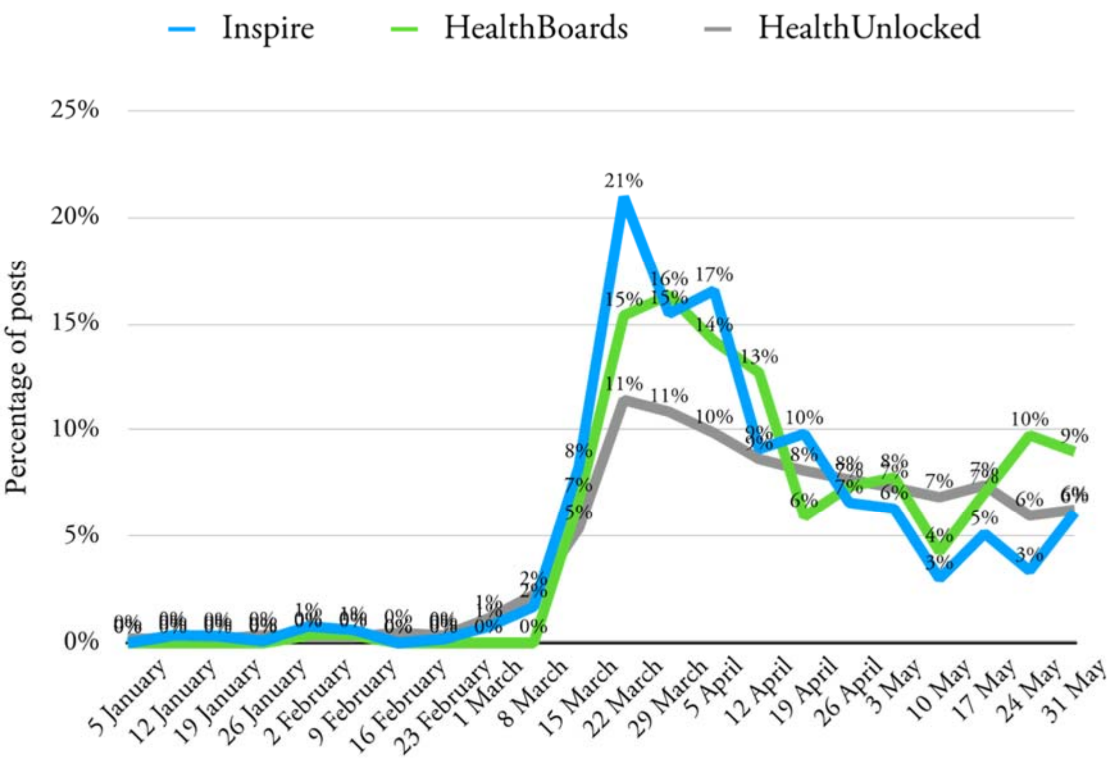

review only

Investigating mental and physical disorders associated with COVID-19 in online health forums: Supplementary Material

Supplementary Figure 3: Maximum number of COVID-19 related threads active in each given week

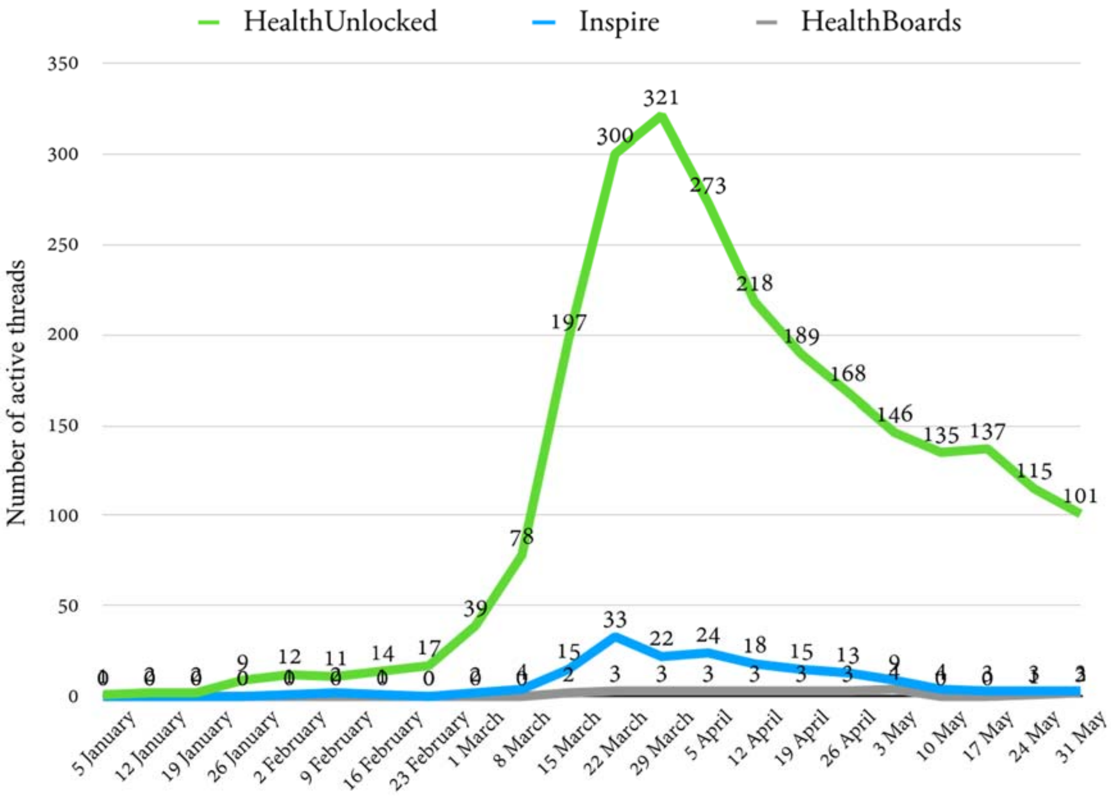

A thread is considered closed and therefore no longer active when there are no further posts from that week within the data collection period (up until 31st May 2020). Note that this does not rule out the possibility of a thread becoming reactivated past this date.

view only

Investigating mental and physical disorders associated with COVID-19 in online health forums: Supplementary Material

Supplementary Figure 4: Proportion of users whose very first post was in a COVID-19 related thread, given weekly

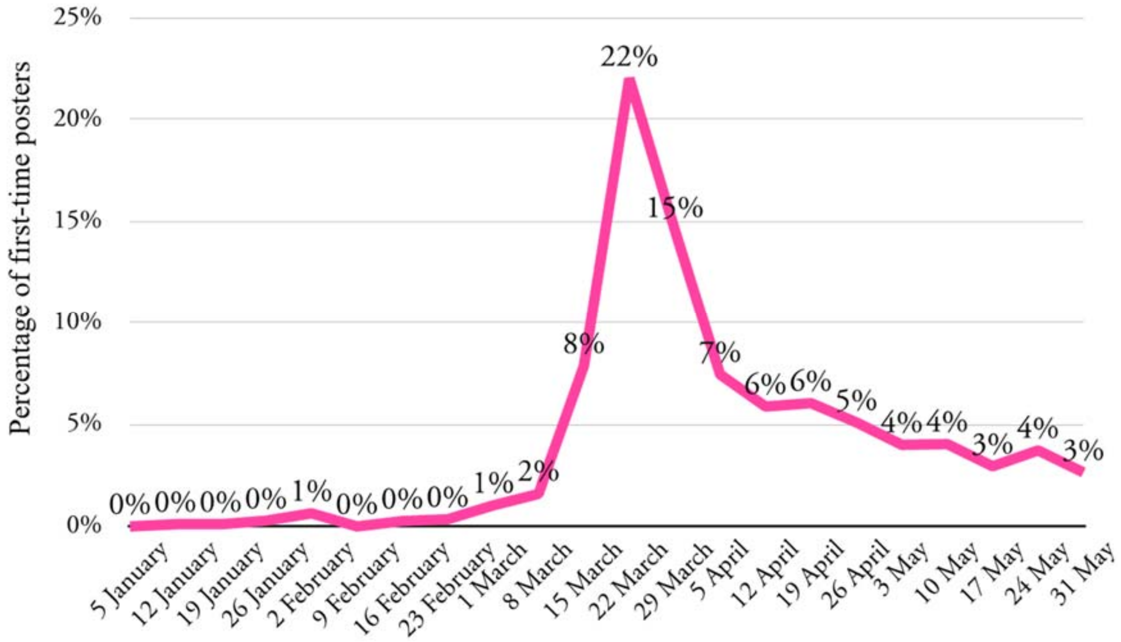

For peer review only

BMJ Open

Analysis of mental and physical disorders associated with COVID-19 in online health forums: a natural language processing study

Journal:	*BMJ Open*
Manuscript ID	bmjopen-2021-056601.R1
Article Type:	Original research
Date Submitted by the Author:	01-Oct-2021
Complete List of Authors:	Patel, Rashmi; King's College London Institute of Psychiatry Psychology and Neuroscience, Department of Psychosis Studies; South London and Maudsley NHS Foundation Trust<br>Smeraldi, Fabrizio; Queen Mary University of London; The Alan Turing Institute<br>Abdollahyan, Maryam; Barts Cancer Institute<br>Irving, Jessica; King's College London Institute of Psychiatry Psychology and Neuroscience, Department of Psychosis Studies<br>Bessant, Conrad; Queen Mary University of London; The Alan Turing Institute
**Primary Subject Heading**:	Health informatics
Secondary Subject Heading:	Intensive care, Mental health
Keywords:	COVID-19, Health informatics < BIOTECHNOLOGY & BIOINFORMATICS, Information technology < BIOTECHNOLOGY & BIOINFORMATICS

SCHOLARONE™
Manuscripts

I, the Submitting Author has the right to grant and does grant on behalf of all authors of the Work (as defined in the below author licence), an exclusive licence and/or a non-exclusive licence for contributions from authors who are: i) UK Crown employees; ii) where BMJ has agreed a CC-BY licence shall apply, and/or iii) in accordance with the terms applicable for US Federal Government officers or employees acting as part of their official duties; on a worldwide, perpetual, irrevocable, royalty-free basis to BMJ Publishing Group Ltd ("BMJ") its licensees and where the relevant Journal is co-owned by BMJ to the co-owners of the Journal, to publish the Work in this journal and any other BMJ products and to exploit all rights, as set out in our licence.

The Submitting Author accepts and understands that any supply made under these terms is made by BMJ to the Submitting Author unless you are acting as an employee on behalf of your employer or a postgraduate student of an affiliated institution which is paying any applicable article publishing charge ("APC") for Open Access articles. Where the Submitting Author wishes to make the Work available on an Open Access basis (and intends to pay the relevant APC), the terms of reuse of such Open Access shall be governed by a Creative Commons licence – details of these licences and which Creative Commons licence will apply to this Work are set out in our licence referred to above.

Other than as permitted in any relevant BMJ Author's Self Archiving Policies, I confirm this Work has not been accepted for publication elsewhere, is not being considered for publication elsewhere and does not duplicate material already published. I confirm all authors consent to publication of this Work and authorise the granting of this licence.

For peer review only

Analysis of mental and physical disorders associated with COVID-19 in online health forums: a natural language processing study

Rashmi Patel[*1,2], Fabrizio Smeraldi[*3,4], Maryam Abdollahyan[5], Jessica Irving[1], and Conrad Bessant[3,4]

1. King's College London (Institute of Psychiatry, Psychology and Neuroscience), London, UK
2. South London and Maudsley NHS Foundation Trust, London, UK
3. Queen Mary University of London, UK
4. The Alan Turing Institute, London, UK
5. Barts Cancer Institute, London, UK

* Equal contribution to authorship

Correspondence to:
Rashmi Patel
E-mail: rashmi.patel@kcl.ac.uk

Manuscript word count: 3,399

Abstract word count: 249

Keywords: COVID-19, comorbidity, real world data, social media, natural language processing

For peer review only

For peer review only - http://bmjopen.bmj.com/site/about/guidelines.xhtml

Investigating mental and physical disorders associated with COVID-19 in online health forums:

Abstract

Objectives: Online health forums provide rich and untapped real-time data on population health. Through novel data extraction and natural language processing (NLP) techniques, we characterise the evolution of mental and physical health concerns relating to the COVID-19 pandemic among online health forum users.

Setting and design: We obtained data from three leading online health forums: HealthBoards, Inspire and HealthUnlocked, from the period 1st January 2020 to 31st May 2020. Using NLP, we analysed the content of posts related to COVID-19.

Primary outcome measures:

(i) Proportion of forum posts containing COVID-19 keywords
(ii) Proportion of forum users making their very first post about COVID-19
(iii) Proportion of COVID-19 related posts containing content related to physical and mental health comorbidities

Results: Data from 739,434 posts created by 53,134 unique users were analysed. A total of 35,581 posts (4.8%) contained a COVID-19 keyword. Posts discussing COVID-19 and related comorbid disorders spiked in early- to mid-March around the time of global implementation of lockdowns prompting a large number of users to post on online health forums for the first time. Over a quarter of COVID-19 related thread titles mentioned a physical or mental health comorbidity.

Conclusions: We demonstrate it is feasible to characterise the content of online health forum user posts regarding COVID-19 and measure changes over time. The pandemic and corresponding public response has had a significant impact on posters' queries regarding mental health. Social media data sources such as online health forums can be harnessed to strengthen population-level mental health surveillance.

Article Summary

Strengths and limitations of this study

1. This study uses a novel approach to examine the content of a large, unstructured online health forum dataset using NLP.
2. Online health forums provide a unique resource of real-world data that represent the lived experiences of people with mental and physical health disorders.
3. It is not yet possible to establish COVID-19 status or whether concerned posters had pre-existing mental or physical health issues, had recovered, or became unwell for the first time during the study period.
4. Online health forums are *help-seeking* forums, which could contribute towards self-selection bias.

For peer review only - http://bmjopen.bmj.com/site/about/guidelines.xhtml
For peer review only

For peer review only

For peer review only - http://bmjopen.bmj.com/site/about/guidelines.xhtml

Introduction

Measures to tackle the COVID-19 pandemic have resulted in unprecedented societal restrictions worldwide. The mental health impacts of these measures and accompanying socioeconomic stressors are likely to be extensive; identifying and quantifying these impacts are now an urgent priority.[1] For example, social distancing restrictions make it harder to maintain regular contact between individuals and their friends and family as well as health and social care professionals. Furthermore, the psychological and emotional burden of the pandemic (and its consequences) may increase risk of relapse or worsen existing mental health disorders. Conversely, mental disorders can increase susceptibility to infections.[2,3]

Real-world data from online resources may be extracted using natural language processing (NLP) techniques to provide automated, population-level health surveillance. These methods can be used to rapidly ascertain discussion related to COVID-19 and associated symptoms and comorbidities. NLP has previously been used to identify medically relevant information from web pages and analyse extracted text.[4,5] Applying these techniques to real world data sources such as social media and online forums may be used to supplement active data collection from participants in prospective observational research. Recent studies have applied this approach to Twitter, Facebook and Reddit data to forecast the emergence of depression and post-traumatic stress disorder,[6] predict depression in the general population,[7] identify mothers at risk of postpartum depression,[8] and investigate suicidal ideation.[9]

While social media platforms such as Twitter, Facebook and Reddit are commonly used, other internet resources such as online health forums have so far been neglected. Online health forums are enriched for health information and receive millions of posts each year, therefore providing untapped reservoirs of healthcare data at population level. Users of online health forums may post information or questions related to a particular health problem and discuss these in written threads with other forum users. Some users are "peer experts" characterised as people with expertise in a particular area of health through lived experience. They may frequently respond to other users' questions and provide practical insights into managing a particular healthcare problem.[10] The typical demographic characteristics of online health forums include a greater proportion of female (78.4%) than male users with 18-34 year old users representing the most frequent age group.[11]

In a recent proof-of-concept study we demonstrated that online health forums can be extracted to detect health discussion trends that correlate with real-life events.[12] Here, we use the same technology to analyse online health forum data discussing mental and physical health problems associated with the COVID-19 pandemic. We use NLP techniques to extract data from online health forum posts related to the COVID-19 pandemic, references to specific comorbid illnesses, and their direct and indirect impacts on mental or physical health.

For peer review only - http://bmjopen.bmj.com/site/about/guidelines.xhtml

Methods

Study Design and Setting

We obtained data from online health forums using NLP. Online forums are discussion websites hosted on the internet where people hold conversations in the form of posted messages. A single conversation is called a thread. Threads are chains of posts identified within a forum by a title and an individual URL. Clicking on the thread title opens the thread which contains one or more posts which may be from the same user who started the thread (i.e. the original poster) or different users who have replied within the thread. In this study, we analysed text data in thread titles and in individual posts within a thread. We analysed posts written in English only. Depending on the forum's settings, users can be anonymous or have to register with the forum to post messages, with most users opting not to use personally identifiable information to register their account. Registration may not be required for read-only access. Most forums recommend that users do not use personally identifiable information when posting. Online health forums specifically cover health topics and offer peer support for various health conditions.

We collected data from three major online health forums posted from 1st January 2020 to 31st May 2020: HealthBoards (www.healthboards.com), Inspire (www.inspire.com) and HealthUnlocked (www.healthunlocked.com). These forums were chosen on the basis that they have global user coverage, include subforums on several aspects of healthcare, have a large user base contributing to regular activity on the forum, and are feasible to extract information from using NLP.

HealthBoards was founded in California, USA in 1997 and offers patient to patient health support. Inspire, founded in 2005, is a US healthcare social network managing online support groups for patients and caregivers. HealthUnlocked is a British online health forum launched in 2011 with a similar offering to HealthBoards and Inspire. Registration and participation in all three forums are free of charge to users.

Analysis Using NLP

Extracting and matching keywords

We extracted the keywords in thread titles and post content using lemmatisation. For flexibility and efficiency, search terms in posts and thread titles were matched using regular expressions that accounted for both inflection and common spelling variants. Supplementary Table 1 provides a key to the regular expression parameters used in the study. Matching was case-insensitive and limited to whole words in the post content and thread title; when matching thread URLs, parts containing words were considered. To prevent spurious matches, words shorter than four letters (e.g. ICU) were considered valid matches only if they were delimited by non-word characters.

Definition of search terms

For peer review only

To investigate the potential impact of COVID-19 on users posting in online health forums, we classified threads and posts using keywords related to the COVID-19 pandemic in conjunction with various groups of case-insensitive keywords relating to a) medical treatment in an intensive care unit or b) physical symptoms as a direct consequence of COVID-19 infection or c) mental health symptoms as a consequence of measures in response to the pandemic.

Search terms used to identify whether a thread or post was related to COVID-19 were 'covid', 'covid-19', 'coronavirus', 'corona', 'sars-cov-2', 'sars-2', 'shielding', 'pandemic*', 'vulnerable', 'quarantine', 'lockdown', 'distancing', 'isolation', 'isolating' where * indicates a wildcard search term.

Table 1 provides the final keywords used to search posts within COVID-19 related threads; the Python coded search terms are provided in Supplementary Tables 2 and 3. We tested the specificity of keywords by searching for matches occurring before 1st January 2020. For threads, these were matches in the title and URL, while for posts, these were matches in the entire text (see *Extracting and matching keywords* below). Term incidence and excluded keywords are provided in Supplementary Table 4.

Data pre-processing

Data obtained from different online health forums come in various formats. We standardised and normalised the data before analysing them. This included normalisation of Unicode strings and whitespace characters, standardisation of date and time, and standardisation of location through the GeoNames.org database.

Analysis of COVID-19 threads to identify changes in COVID-19 related user activity and physical and mental health associations over time

We identified the users contributing to a COVID-19 related thread in a given week. We then retrieved all the other posts made by the same authors in the previous, same and subsequent calendar weeks. We scanned such posts for physical symptom, mental health symptom or intensive care keywords as defined in Table 1, and recorded whether each of these topics was mentioned by the author during the time window. We performed this analysis to establish variations in the prevalence of concerns relating to physical symptom, mental health symptom and intensive care keywords over the course of the pandemic during 2020. Weekly counts were measured each Sunday for the previous week.

Analysis of thread titles

We inspected thread titles to identify how many mentioned a comorbidity in the title. We searched for terms related to autoimmune disorders, mental disorders or worry, cancer, cardiovascular problems or stroke, and diabetes as listed in Table 2; the Python coded search terms are provided in Supplementary Table 5.

Analysis of first-time posters in a COVID-19 related thread

We analysed the first ever post published by a user to determine the proportion of first-time posters who started out by contributing to a COVID-19 related thread. We performed this

For peer review only - http://bmjopen.bmj.com/site/about/guidelines.xhtml

analysis to determine the degree to which new users were motivated to make their first post in relation to the COVID-19 pandemic and how this varied over time during 2020.

Implementation and computation

All descriptive analyses were performed using bespoke software written in Python. An outline of the coding approach employed is included in the Supplementary Material.

Ethics and Data Sharing

The study was independently reviewed by the Queen Mary Ethics of Research Committee (QMERC) and received an exemption for ethical approval. All data were analysed on Queen Mary University of London (QMUL) IT facilities, which employs a two-layer security model as per their security policy. We consulted and adhered to internet research guidelines from the Association of Internet Researchers [13] and the British Psychological Society (BPS) [14] to inform study development.

Given licensing and privacy issues, it is not possible to publicly release the aggregate dataset generated from the three online health forums investigated. However, we welcome collaboration with other researchers and healthcare policy makers. Anyone interested in accessing the aggregate data and data analysis code should contact the guarantor (f.smeraldi@qmul.ac.uk).

Patient and public involvement

Patients and the public were not involved in the design or conduct of the study.

For peer review only

Results

Related posts and active threads

HealthUnlocked was the most frequently used forum accounting for 97% of overall posts and 97% of posts mentioning COVID-19 in the thread title or post content during the study period (Table 3).

Weekly post count for HealthUnlocked peaked in mid-March. Post count for Inspire declined sharply in the last two weeks of March. Post count for HealthBoards declined slowly across the entirety of the observation period (Supplementary Figure 1).

Across all three forums, there were a total of 3,342 threads containing a COVID-19 keyword within the thread title or URL. These contained a total of 44,894 posts during the study period (1st January 2020 to 31st May 2020). A total of 35,581 posts (whether in COVID-19 related threads or otherwise) contained a COVID-19 keyword during the study period. The proportion of posts containing COVID-19 keywords increased rapidly across all forums in early March (Supplementary Figures 2 and 3), corresponding with the World Health Organisation's declaration of COVID-19 as a pandemic on 11th March 2020. The smaller online forums (Inspire and HealthBoards) had a greater peak in percentage of total posts containing COVID-19 keywords. The total number of posts containing COVID-19 search terms declined from mid-April onwards.

For quite a long period, most posts about COVID-19 (over 90% at the beginning of the observation period, and remaining above 50% until the week ending 29th March) were written by users who had not yet posted on the topic. By the end of the observation period, the percentage of weekly posts in COVID-19 threads written by new entrants to the discussion reduced to a still quite sizeable 30%. While many of these users may have posted before on the forum about other topics, Supplementary Figure 4 presents the proportion of posters whose very first post to a forum appeared in a COVID-19 related thread. This figure peaked above 20% in the week ending 22nd March. Considering that these forums have a very broad spectrum, this is a remarkably high fraction. It includes both new joiners and users who were previously silent members of the forums, possibly for a long time (so-called "lurkers"), and who may have been spurred into a more active role by the pandemic.

Thread title analysis

Over a quarter of COVID-19 related thread titles mentioned another condition of interest (Table 4). After cancer and autoimmune diseases, mental health represented a major area of concern for online health forum users posting about COVID-19, comparable to respiratory and circulatory diseases (Table 2). Around 0.5% of thread titles mentioned two or more comorbidities.

User analysis

Posts in threads related to COVID-19 were analysed to determine the number of users contributing in each given week. For each active user, all posts in the previous, same and

For peer review only - http://bmjopen.bmj.com/site/about/guidelines.xhtml

following calendar weeks were scanned irrespective of thread for mentions of physical symptom, mental health symptom or intensive care keywords. The number of active users mentioning each of these concerns peaked in the week ending 22nd March and subsequently declined but still remained elevated above the January baseline. In particular, users discussing mental health outnumbered users mentioning the other topics (Figure 1).

For peer review only

Discussion

Using a novel technique to analyse data from online health forums, we found a marked increase in posts related to COVID-19 across the observation period of 1st January 2020 to 31st May 2020. The frequency of these posts increased rapidly in early March 2020 corresponding with the World Health Organisation's declaration of COVID-19 as a pandemic.

During this period, we found mental health symptom keywords were most frequently mentioned by authors of COVID-19 related posts (either contextually or in separate messages), followed by physical symptoms and intensive care keywords, suggesting that the pandemic and public health response to it has had a significant impact on posters' concerns regarding mental health. The marked increase in mental health symptom related posts in early March, when the WHO declared the COVID-19 pandemic, correlates with preliminary worldwide data that show increases in anxiety and depression in response to the outbreak.

The mental health impacts of COVID-19 and associated physical distancing restrictions are likely to be extensive and wide-reaching. There is a growing body of evidence supporting the neuropsychiatric effects of coronavirus infections.[15] Restrictions fuel socioeconomic stressors such as unemployment, loneliness and financial burden, which are all implicated in the development of mental ill health.[16] Increased rates of bereavement, newfound caring responsibilities and interruptions to education are likely to be particularly stressful to children and young adults.[17]

A preliminary survey of 3,545 German respondents found evidence of substantial mental health burden from travel and physical distancing restrictions, including increased levels of stress, anxiety, depressive symptoms, sleep disturbance and irritability.[18] Worsening mental health has been confirmed in samples with both pre- and post-pandemic information for direct comparison: The Avon Longitudinal Study of Parents and Children (ALSPAC) study found probable anxiety disorder doubled compared to pre-pandemic sizes (26% vs 13%) and lower wellbeing, particularly in young people, women and those with pre-existing conditions.[19] The literature on social media mining for COVID-19 mental health related trends is limited. A study analysing sentiment evolution trends of four emotions across Twitter – fear, anger, sadness, joy – has been able to identify developing shared distress, and topics of interest relating to those emotions.[20]

Our findings also suggest that mental and physical health concerns documented in online forum posts have levelled off following their peak in March 2020 but their rates continued to remain above pre-pandemic levels. The number of users active in COVID-19 threads who also wrote posts concerning mental health symptoms reduced from their peak in March of 1,355 (per week) to 253 by the end of the observation period (compared to a mean number of 30 per week in January), suggesting that as time went on most users had begun to adjust to the consequences of the pandemic. Furthermore, the increased engagement of users on online health forums could be related to the restrictions on travel and in-person interaction during the pandemic leading to a shift to social engagement through online media. Other NLP studies have also

For peer review only - http://bmjopen.bmj.com/site/about/guidelines.xhtml

identified a similar trend. An analysis of 10 million Google searches within the United States found large shifts in mental health symptom searches linked to stay-at-home orders in the United States across the week commencing 16th March 2020.[21] Searches for topics related to anxiety, negative thoughts about oneself and the future, insomnia and suicidal ideation dramatically increased prior to stay-at-home orders, levelling off upon the announcement of stay-at-home orders. These patterns were relatively unique to searches for mental health related information and not physical conditions.

We used keywords related to intensive care treatment in NLP models reflecting the potential for COVID-19 to lead to serious illness requiring intensive care treatment. We found that threads containing content related to intensive care treatment increased in frequency during the pandemic. Follow-up data in more recent studies has highlighted the potential for COVID-19 to be associated with prolonged, multisystem involvement associated with significant disability[22] with considerable reduction in functioning and quality of life among individuals who have received intensive care treatment.[23] A significant proportion of people who have received intensive care treatment for COVID-19 have post-intensive care syndrome characterised by physical and cognitive impairments, and problems with mental health.[24] This could explain the increasing rates of threads related to intensive care treatment due to forum users' concerns of the potential need for intensive care treatment, raising questions about family members or friends currently receiving intensive care treatment, or in relation to problems faced after being discharged from the intensive care setting.

Over the entire period, on average 4% of first-time posters (over 20% in the peak period) made their very first contribution to the forum in a COVID-19 related thread. Furthermore, 77% of COVID-19 threads were started by users who had never posted about the topic before, and chose to start out by creating their own thread. A certain degree of motivation is required to take someone to the point of making that first post on a forum, and also for starting a thread; our finding suggests that the pandemic is driving users to engage more actively in community forum services in times of uncertainty.

Strengths and weaknesses

Online health forums are an important source of real-world, real-time, population-level data on people living through the COVID-19 pandemic. Online health forums also afford users anonymity to discuss aspects of their experience they might otherwise have been embarrassed or fearful to disclose in identifiable forms of social media. We have demonstrated that it is possible to automate information extraction from these posts using natural language processing, providing access to a rich reservoir of previously untapped real-world data from health-specific online resources.

Our approach was able to automatically extract data from a large sample of over 53,000 unique users at a fraction of the cost of previous approaches that have relied on social media individual participant recruitment and manual review of posts generating sample sizes in the low hundreds.[7] Some studies screened users on Twitter via depression symptom questionnaires

For peer review only

For peer review only - http://bmjopen.bmj.com/site/about/guidelines.xhtml

and used their tweets to train depression onset classifiers.[6,25] Analogous approaches have been used with Facebook data.[8]

Our study has some limitations. At present it is difficult to establish whether concerned posters have pre-existing mental or physical health issues, have experienced confirmed COVID-19 illness themselves, are recovered, or have become unwell for the first time. Online health forums are help-seeking communities; this introduces self-selection bias in which individuals from disadvantaged backgrounds who do not have IT equipment/network connection to access online resources are under-represented and our results are therefore not generalisable to the entire population. Furthermore, as these forums have worldwide coverage we cannot isolate trends to one geographic region. However, future work could utilise the location data (see *Data pre-processing* in Methods) to explore this avenue. We analysed data up to 31st May 2020 providing a relatively short period of follow-up following the onset of the COVID-19 pandemic. Future studies examining a longer period of follow-up would help to determine if the increase in activity and content related to COVID-19 and comorbid health problems persisted. As we analysed anonymised data, we were unable to investigate the demographic characteristics of the forum users in our study to determine whether they were representative of the wider population.

Conclusions and future research

Publicly accessible sources of real-world data, such as online health forums analysed in this study, can strengthen population-level physical and mental health surveillance and provide a rapid and inexpensive means to inform public healthcare policy. We found that the majority of posts in online forum data related to COVID-19 concerned features related to mental health and that the peak in frequency of posts corresponded with the early phase of the pandemic, indicating the significant impact of COVID-19 on the mental health of susceptible populations.

As the pandemic evolves, further research using online forum data could improve our understanding of the long-term consequences of COVID-19 infection [26] and the longer-term socioeconomic consequences of travel and physical distancing restrictions that have been employed in many countries to manage viral transmission.[27,28] Analysis of real-world data, including social media and online health forums, could provide a useful insight into attitudes and perceptions towards novel therapeutics. This will be crucial to maximising continued uptake of effective preventative approaches such as mask-wearing, physical distancing, ventilation, hygiene measures and vaccines.

For peer review only - http://bmjopen.bmj.com/site/about/guidelines.xhtml

Competing interests: All authors have completed the ICMJE uniform disclosure form and declare: RP has received funds from Janssen, Induction Healthcare and Holmusk outside the current study. The other authors declare no competing interests.

Contributorship statement: The study was conceived by RP and FS. Data extraction and statistical analysis were performed by FS, MA and CB. Reporting of findings were carried out by RP and JI. All authors (RP, FS, MA, JI and CB) contributed to study design, manuscript preparation and approved the final version. FS is guarantor.

Ethics approval: Data for this study were drawn from publicly available online health forums and extracted in aggregate form for secondary data analysis rather than at individual user level. The study was independently reviewed by the Queen Mary Ethics of Research Committee (QMERC) and received an exemption for ethical approval. The data were analysed using the computing infrastructure based at Queen Mary University of London (QMUL) which employs a two-layer security model to maintain data privacy. QMUL is registered as a data controller with the Information Commissioner's Office (ICO; registration number: Z5507327), which covers all research activities undertaken at the university.

Source of funding: RP has received support from a Medical Research Council (MRC) Health Data Research UK Fellowship (MR/S003118/1), a National Institute for Health Research (NIHR) Advanced Fellowship (NIHR301690) and a Starter Grant for Clinical Lecturers (SGL015/1020) supported by the Academy of Medical Sciences, The Wellcome Trust, MRC, British Heart Foundation, Arthritis Research UK, the Royal College of Physicians and Diabetes UK. FS and CB were funded by an Alan Turing Institute (ATI) Fellowship and by an EPSRC COVID-19 Rapid Response Impact Acceleration Fund. Computational resources were funded by a Microsoft Azure Sponsorship through the ATI.

Role of funder: The views expressed are those of the authors and not necessarily those of the funders. The funders had no role in the design and conduct of the study; collection, management, analysis, and interpretation of the data; preparation, review, or approval of the manuscript; and decision to submit the manuscript for publication.

Data sharing: Given licensing and privacy issues, it is not possible to release the dataset generated from the online health forums investigated in this study. However, we welcome collaboration with other researchers and healthcare policy makers. Anyone interested in accessing the aggregate data and data analysis code should contact the guarantor (f.smeraldi@qmul.ac.uk).

For peer review only

References

1 Holmes EA, O'Connor RC, Perry VH, *et al.* Multidisciplinary research priorities for the COVID-19 pandemic: a call for action for mental health science. The Lancet Psychiatry. 2020;**7**:547–60. doi:10.1016/S2215-0366(20)30168-1

2 Yao H, Chen J-H, Xu Y-F. Patients with mental health disorders in the COVID-19 epidemic. 2020. doi:10.1016/S2215-0366(20)30090-0

3 Seminog OO, Goldacre MJ. Risk of pneumonia and pneumococcal disease in people with severe mental illness: English record linkage studies. *Thorax* Published Online First: 2013. doi:10.1136/thoraxjnl-2012-202480

4 Landers RN, Brusso RC, Cavanaugh KJ, *et al.* A primer on theory-driven web scraping: Automatic extraction of big data from the internet for use in psychological research. *Psychol Methods* 2016;**21**:475–92. doi:10.1037/met0000081

5 Cambria E, White B. Jumping NLP curves: A review of natural language processing research. IEEE Comput. Intell. Mag. 2014;**9**:48–57. doi:10.1109/MCI.2014.2307227

6 Reece AG, Reagan AJ, Lix KLM, *et al.* Forecasting the onset and course of mental illness with Twitter data. *Sci Rep* 2017;**7**:1–11. doi:10.1038/s41598-017-12961-9

7 De Choudhury M, Gamon M, Counts S, *et al.* Predicting Depression via Social Media. 2013.

8 De Choudhury M, Counts S, Horvitz E. Predicting postpartum changes in emotion and behavior via social media. In: *Conference on Human Factors in Computing Systems - Proceedings*. New York, New York, USA: : ACM Press 2013. 3267–76. doi:10.1145/2470654.2466447

9 De Choudhury M, Kiciman E, Dredze M, *et al.* Discovering shifts to suicidal ideation from mental health content in social media. In: *Conference on Human Factors in Computing Systems - Proceedings*. Association for Computing Machinery 2016. 2098–110. doi:10.1145/2858036.2858207

10 Vydiswaran VGV, Reddy M. Identifying peer experts in online health forums. *BMC Med Inform Decis Mak* 2019;**19**:68. doi:10.1186/s12911-019-0782-3

11 Sadah SA, Shahbazi M, Wiley MT, *et al.* A Study of the Demographics of Web-Based Health-Related Social Media Users. *J Med Internet Res* 2015;**17**:e194. doi:10.2196/jmir.4308

12 Abdollahyan M, Smeraldi F, Patel R, *et al.* Investigating Comorbidity of Mental and Physical Disorders in On-line Health Forums. Published Online First: 2020. doi:10.1145/3378184.3378195

13 Franzke AS, Bechmann A, Zimmer M, *et al.* Internet Research: Ethical Guidelines 3.0. 2020.

14 British Psychological Society. British Psychological Society Ethics Guidelines for Internet-mediated Research. *Br Psychol Soc* Published Online First: 2017. doi:INF206/1.2013

15 Rogers JP, Chesney E, Oliver D, *et al.* Psychiatric and neuropsychiatric presentations associated with severe coronavirus infections: a systematic review and meta-analysis with comparison to the COVID-19 pandemic. *The Lancet Psychiatry* 2020;**7**:611–27. doi:10.1016/S2215-0366(20)30203-0

16 Kawohl W, Nordt C. COVID-19, unemployment, and suicide. The Lancet Psychiatry. 2020;**7**:389–90. doi:10.1016/S2215-0366(20)30141-3

17 McManus S, Bebbington P, Jenkins R, *et al.* Mental health and wellbeing in England. 2016.

18 Jung S, Kneer J, Tillmann HC, *et al.* The German COVID-19 Survey on Mental Health: Primary Results. doi:10.1101/2020.05.06.20090340

19 F Kwong AS, Pearson M, Adams J, *et al.* Mental health during the COVID-19 pandemic in two longitudinal UK population cohorts 1. doi:10.1101/2020.06.16.20133116

20 Lwin MO, Lu J, Sheldenkar A, *et al.* Global Sentiments Surrounding the COVID-19 Pandemic on Twitter: Analysis of Twitter Trends. doi:10.2196/19447

21 Jacobson NC, Lekkas D, Price G, *et al.* Flattening the Mental Health Curve: COVID-19 Stay-at-Home Orders Are Associated With Alterations in Mental Health Search Behavior in the United States. *JMIR Ment Heal* Published Online First: 2020. doi:10.2196/19347

22 Davis HE, Assaf GS, McCorkell L, *et al.* Characterizing long COVID in an international cohort: 7 months of symptoms and their impact. *EClinicalMedicine* 2021;**38**.

For peer review only

doi:10.1016/j.eclinm.2021.101019

23 Rousseau A-F, Minguet P, Colson C, *et al.* Post-intensive care syndrome after a critical COVID-19: cohort study from a Belgian follow-up clinic. *Ann Intensive Care* 2021;**11**:118. doi:10.1186/s13613-021-00910-9

24 Nakanishi N, Liu K, Kawakami D, *et al.* Post-Intensive Care Syndrome and Its New Challenges in Coronavirus Disease 2019 (COVID-19) Pandemic: A Review of Recent Advances and Perspectives. J. Clin. Med. . 2021;**10**. doi:10.3390/jcm10173870

25 Tsugawa S, Kikuchi Y, Kishino F, *et al.* Recognizing depression from twitter activity. In: *Conference on Human Factors in Computing Systems - Proceedings*. New York, New York, USA: : Association for Computing Machinery 2015. 3187–96. doi:10.1145/2702123.2702280

26 Mahase E. Covid-19: What do we know about 'long covid'? BMJ. 2020;**370**. doi:10.1136/bmj.m2815

27 Rossi R, Socci V, Talevi D, *et al.* COVID-19 Pandemic and Lockdown Measures Impact on Mental Health Among the General Population in Italy. *Front Psychiatry* 2020;**11**. doi:10.3389/fpsyt.2020.00790

28 Veer IM, Riepenhausen A, Zerban M, *et al.* Psycho-social factors associated with mental resilience in the Corona lockdown. doi:10.31234/OSF.IO/4Z62T

For peer review only

Figure 1 legend:

Number of users making posts in threads related to COVID-19 which included physical symptoms, mental health symptoms or intensive care keywords

For peer review only

Table 1: Physical health, mental health and intensive care keywords used to search threads and posts

**Physical COVID-19 symptoms**	chest pain, smell, taste, dry cough, anosmia, breath*
**Mental health symptoms**	worried, worry, worrying, worries, anxious, anxiety, feel(s) low, feeling low, depression, depressed, low mood
**Intensive care terms**	itu, icu, intensive care, intubation, intubated, ventilated, ventilator, c-pap, ecmo, membrane oxygenation

Table 2. Keywords applied to threads containing COVID-19 search terms to investigate physical and mental health diagnoses potentially associated with the COVID-19 pandemic

**Comorbid condition**	**Search terms**
Cardiovascular/stroke	heart, infarct*, bypass, stent, coronary, ablation, atrial fibrillation, arrhythmia, aortic, cardio*, blockers, cardiomyopathy, statin(s), pressure, valve, pacemaker, stroke, ischaemia, ischaemic, anticoag*, anticoagulants, xarelto, apaxiban, rivaroxiban, dabigatran
Cancer	cancer, chemo, chemotherapy, tumours, melanoma, leukaemia, radiation, radiotherapy
Respiratory diseases	asthma, asthmatic, copd
Mental disorders	anxiety, anxious, depression, depressive, psychosis, psychotic, bipolar, schizophrenia, schizoaffective, ocd, ptsd
Autoimmune diseases	crohns, psoriasis, immunosuppress(ant), lupus, multiple sclerosis, ms, auto-immune
Diabetes	diabetes, mellitus, insulin, humira, remicade, metformin

* denotes a wildcard search

For peer review only - http://bmjopen.bmj.com/site/about/guidelines.xhtml

Table 3: Number of users and posts retrieved from selected online health forums from 1st January 2020 to 31st May 2020

Website	Number of users[a]	Number of posts	Number (%) of posts mentioning COVID-19
HealthUnlocked *(www.healthunlocked.com)*	47,999	718,103	34,657 (4.8%)
HealthBoards *(www.healthboards.com)*	601	3,477	127 (3.7%)
Inspire *(www.inspire.com)*	4,534	17,854	797 (4.5%)
**Total**	**53,134**	**739,434**	**35,581 (4.8%)**

[a] A user is defined as anyone who posted within the data collection period

Table 4: Number and proportion of COVID-19 related threads and posts contained within COVID-19 related threads that mention a comorbidity

Comorbidity	Number of threads, n (%)[a]	Number of posts contained within threads, n (%)[b]
No condition of interest	2,340 (70.0%)	34,201 (76.2%)
Autoimmune disorders	254 (7.6%)	2,739 (6.1%)
Cancer	223 (6.7%)	2,114 (4.7%)
Respiratory	150 (4.5%)	1,905 (4.2%)
Cardiovascular / Stroke	133 (4.0%)	1,583 (3.5%)
Diabetes	45 (1.3%)	295 (0.7%)
Mental disorders	215 (6.4%)	2,243 (5.0%)

[a] Percentage is calculated as number of mentions of comorbidities/ total threads (N = 3,342).
[b] Percentage is calculated as number of posts / total posts (N = 44,894)
Note: Percentages do not add up to 100% because some threads contained mentions of more than one comorbidity

For peer review only

For peer review only - http://bmjopen.bmj.com/site/about/guidelines.xhtml

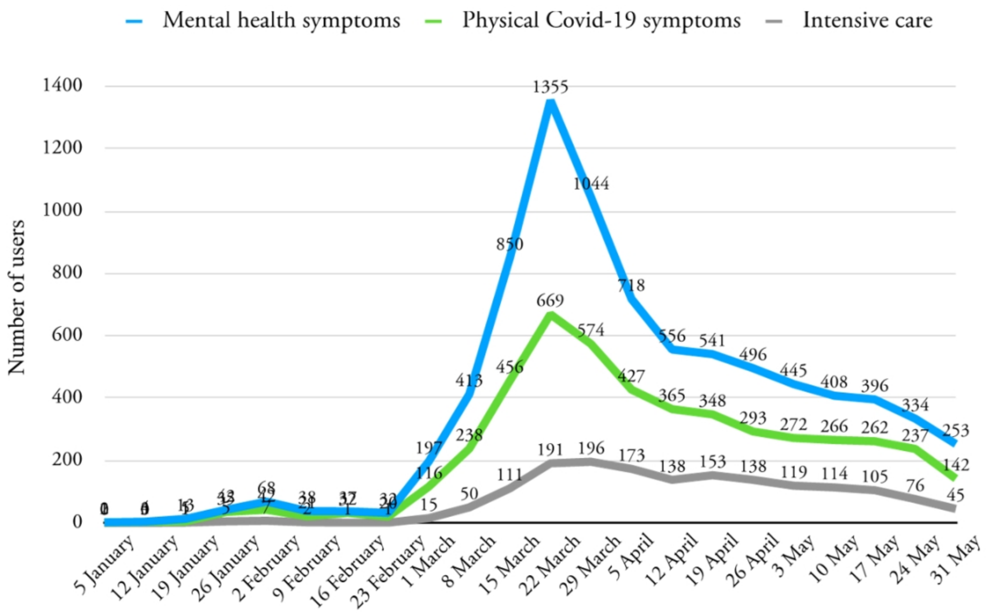

Figure 1: Number of users making posts in threads related to COVID-19 which included physical symptoms, mental health symptoms or intensive care keywords

108x67mm (300 x 300 DPI)

Investigating mental and physical disorders associated with COVID-19 in online health forums

Supplementary Material

Methods - Search Terms and Keywords

Supplementary Table 1. Key to regular expressions

[]	One of the characters inside the bracket
+	One or more occurrences of previous character
*	Zero or more occurrences of previous character
?	Zero or one occurrence of previous character
\w	Any alphanumeric character
\s	Any whitespace character

Examples:

- 'auto[_\s-]?immune' will match all of "autoimmune", "auto-immune" and "auto immune".
- 'psycho[st]i[sc]' will match both "psychotic" and "psychosis"
- 'infarct\w*' will match "infarct", "infarcts", "infarction", "infarctions" and "infarcted"

Matching is case-insensitive. When matching post content or thread titles, keywords have to appear as separate whole words; this requirement is lifted when matching thread URLs. To reduce spurious matches, keywords up to three letters long have to appear in URLs surrounded by underscores (_) or other non-alphanumeric characters in order to be counted as a match.

Supplementary Table 2. Terms used to determine whether a thread or a post was about Covid; these were applied to both thread titles/URLs and post content

**Covid terms**	'covid', 'covid-?19', 'coronavirus', 'corona', 'sars-?cov-?2', 'sars-?2'<br>'shielding', 'pandemi\w*', 'vulnerable', 'quarantined?', 'lockdown', 'distancing', 'isolation', 'isolating'
**Discarded Covid terms**	'challenging[_\s]times?', 'difficult[_\s]times?', 'hard[_\s]times?'

For peer review only

Investigating mental and physical disorders associated with COVID-19 in online health forums: Supplementary Material

Supplementary Table 3. Terms used to search post content only

**Mental health symptoms**	'worried', 'worry', 'worrying', 'worries', 'anxious', 'anxiety', 'feel[_\s]low', 'feeling[_\s]low', 'depression', 'depressed', 'low[_\s]mood'
**Intensive care terms**	'itu', 'icu', 'intensive[_\s]care', 'intubation', 'intubated', 'ventilated', 'ventilator', 'c-?pap', 'ecmo', 'membrane[_\s]oxygenation'
**Physical Covid symptoms**	'chest[_\s]pain', 'smell', 'taste', 'dry[_\s]cough', 'anosmia', 'breathe?', 'breathing'

Supplementary Table 4. Term incidence of keywords used to scan thread titles/URLs for the observation period (January 1st – May 31st 2020) vs a control period (September 1st – December 31st 2019)

**Keyword**	**Term incidence**		**Included/Excluded?**
	**Observation period (Jan 1st – May 31st 2020)**	**Control period (Sep 1st – December 31st 2019)**	
**covid**	1395	0	Included
**covid-?19**	1205	0	Included
**coronavirus**	859	0	Included
**corona**	1140	14	Included
**sars-?cov-?2**	7	0	Included
**sars-?2**	0	0	Included
**shielding**	202	0	Included
**pandemi\w***	79	0	Included
**vulnerable**	120	3	Included
**quarantined?**	54	0	Included
**lockdown**	207	1	Included
**distancing**	82	0	Included
**isolation**	248	2	Included
**isolating**	82	0	Included
**challenging[_\s]times?**	7	0	Excluded
**difficult[_\s]times?**	13	5	Excluded
**hard[_\s]times?**	22	10	Excluded

For peer review only

Investigating mental and physical disorders associated with COVID-19 in online health forums: Supplementary Material

Supplementary Table 5. Keywords applied to thread titles/URLs together with Covid terms to determine threads concerning Covid comorbidities

Comorbid condition	Search terms
Heart/Stroke	'heart', 'infarct\w*', 'bypass', 'stent', 'coronary', 'ablation', 'a-?fib', 'af', 'arrhythmia', 'aortic', 'cardio', 'blockers', 'cardiom[yi]opathy', 'statins?', 'pressure', 'valve', 'pacemaker', 'stroke', 'ischa?emia', 'ischa?emic', 'anticoag', 'anticoagulants?', 'xarelto', 'apaxiban', 'rivaroxiban', 'dabigatran'
Cancer	'cancer', 'chemo', 'chemotherapy', 'tumou?rs?', 'melanoma', 'leuka?emia', 'radiation', 'radiotherapy'
Respiratory diseases	'asthma', 'asthmatic', 'copd'
Mental (health) disorders	'anxiety', 'anxious', 'depression', 'depressive', 'psycho[st]i[sc]', 'bipolar', 'schizophrenia', 'schizoaffective', 'ocd', 'ptsd'
Autoimmune diseases	'crohns', 'psoriasis', 'immunosuppress\w+', 'lupus', r'multiple[_\s]sclerosis', 'ms', 'auto[_\s-]?immune'
Diabetes	'diabet\w+', 'mellitus', 'insulin', 'humira', 'remicade', 'metformin'

For peer review only

Investigating mental and physical disorders associated with COVID-19 in online health forums: Supplementary Material

For peer review only

Investigating mental and physical disorders associated with COVID-19 in online health forums: Supplementary Material

Methods - Coding Approach

Figure 1

```
# counting users who post in threads about COVID-19 and also express a specific concern, i.e. mention keywords from a list of search terms (e.g. mental_symptom_terms) for each given week in time period
DEFINE count_user_concerns_week
FOR each user
    FOR each week in date_range
        IF user posted in a COVID-19 thread during week
            DEFINE sliding_window ← (previous, current and next calendar week)
            FOR each post by user
                IF date of post in sliding_window and post content matches regex(keywords)
                    INCREMENT count_user_concerns_week
                    BREAK   # exit innermost loop
                ENDIF
            ENDFOR
        ENDIF
    ENDFOR
ENDFOR
```

For peer review only

Investigating mental and physical disorders associated with COVID-19 in online health forums: Supplementary Material

Tables 1 and 2

```
DEFINE
websites                                    # list of online health forums
covid_terms                                 # list of COVID-19 terms
pandemic_terms                              # list of pandemic terms
symptom_terms                               # list of physical COVID-19 symptoms
intensive_care_terms                        # list of intensive care terms
mental_symptom_terms                        # list of mental health symptoms
mental_drug_terms                           # list of drugs for mental illnesses
comorbidity_terms                           # list of comorbid conditions
date_range ← January 1st – May 31st 2020    # time period

FUNCTION regex
    INPUT list of keywords from a list of search terms
    OUTPUT compiled regex for text containing any of the keywords in the list
ENDFUNCTION
```

Table 3, Supplementary Figure 1

```
# counting posts for each given week in time period
DEFINE count_post_week
FOR each website in websites
    FOR each post on website
        FOR week in date_range
            IF date of post is in week
                INCREMENT count_post_week
            ENDIF
        ENDFOR
    ENDFOR
ENDFOR

# computing total number of posts
COMPUTE count_post_all as sum of count_post_week over date_range
```

For peer review only

Investigating mental and physical disorders associated with COVID-19 in online health forums: Supplementary Material

Supplementary Figure 2

```
# counting posts mentioning keywords from a list of search terms (e.g. covid_terms) for each given week in time period
DEFINE count_relevant_post_week
FOR each website in websites
    FOR each post on website
        FOR week in date_range
            IF date of post is in week AND post content matches regex(keywords)
                INCREMENT count_relevant_post_week
            ENDIF
        ENDFOR
    ENDFOR
ENDFOR

# Note: To assess the specificity of search terms, we looked for matches before 1st January 2020, i.e. similar to the above with date_range ← < 1st January 2020

# computing relative frequency of posts mentioning keywords from a list of search terms (e.g. covid_terms) for each given week in time period
COMPUTE ratio of count_relevant_post_week to count_post_week
```

For peer review only

Investigating mental and physical disorders associated with COVID-19 in online health forums: Supplementary Material

Supplementary Figure 3

```
# counting active threads about COVID-19 for each given week in time period
DEFINE count_covid_thread
FOR each website in websites
        FOR each thread on website
                IF thread title or URL matches regex(keywords from covid_terms)
                        # date of first and last posts, respectively
                        DEFINE active_period ← (thread.start_date, thread.end_date)
                        FOR week in date_range
                                IF week is in active_period     # thread is active
                                        INCREMENT count_covid_thread
                                ENDIF
                        ENDFOR
                ENDIF
        ENDFOR
ENDFOR
```

Supplementary Figure 4

```
# counting users who post for the first time for each given week in time period
DEFINE count_first_time_user
FOR each user
        FOR week in date_range
                IF date of user's first post is in week
                        INCREMENT count_first_time_user
                ENDIF
        ENDFOR
ENDFOR

# counting users whose first post is about COVID-19 for each given week in time period
DEFINE count_covid_first_time_user
FOR each user
        FOR week in date_range
                IF date of user's first post is in week AND post content matches
        regex(keywords from covid_terms)
                        INCREMENT count_covid_first_time_user
                ENDIF
        ENDFOR
ENDFOR

# computing percentage of users whose first post is about COVID-19 for each given week in
time period
COMPUTE percentage of count_covid_first_time_user in count_first_time_user
```

For peer review only

Investigating mental and physical disorders associated with COVID-19 in online health forums: Supplementary Material

Table 4

```
# counting threads about a comorbid condition within threads about COVID-19
DEFINE count_comorbid_condition_thread
FOR each COVID-19 thread  # see code for Supplementary Figure 3
	IF thread title or URL matches regex(keyword (i.e. condition) from comorbidity_terms)
		INCREMENT count_comorbid_condition_thread
	ENDIF
ENDFOR

# computing ratio of threads about a comorbid condition to threads about COVID-19
COMPUTE ratio of count_comorbid_condition_thread to sum of count_covid_thread over date_range

# computing number of posts in threads about a comorbid condition
COMPUTE count_comorbid_condition_post as sum of number of posts in threads about a comorbid condition

# computing ratio of posts in threads about a comorbid condition to posts in threads about COVID-19
COMPUTE ratio of count_comorbid_condition_post to sum of number of posts in threads about COVID-19
```

For peer review only

Investigating mental and physical disorders associated with COVID-19 in online health forums: Supplementary Material

Supplementary Figure 1: Weekly number of posts on any topic in the period January 2020 to May 2020

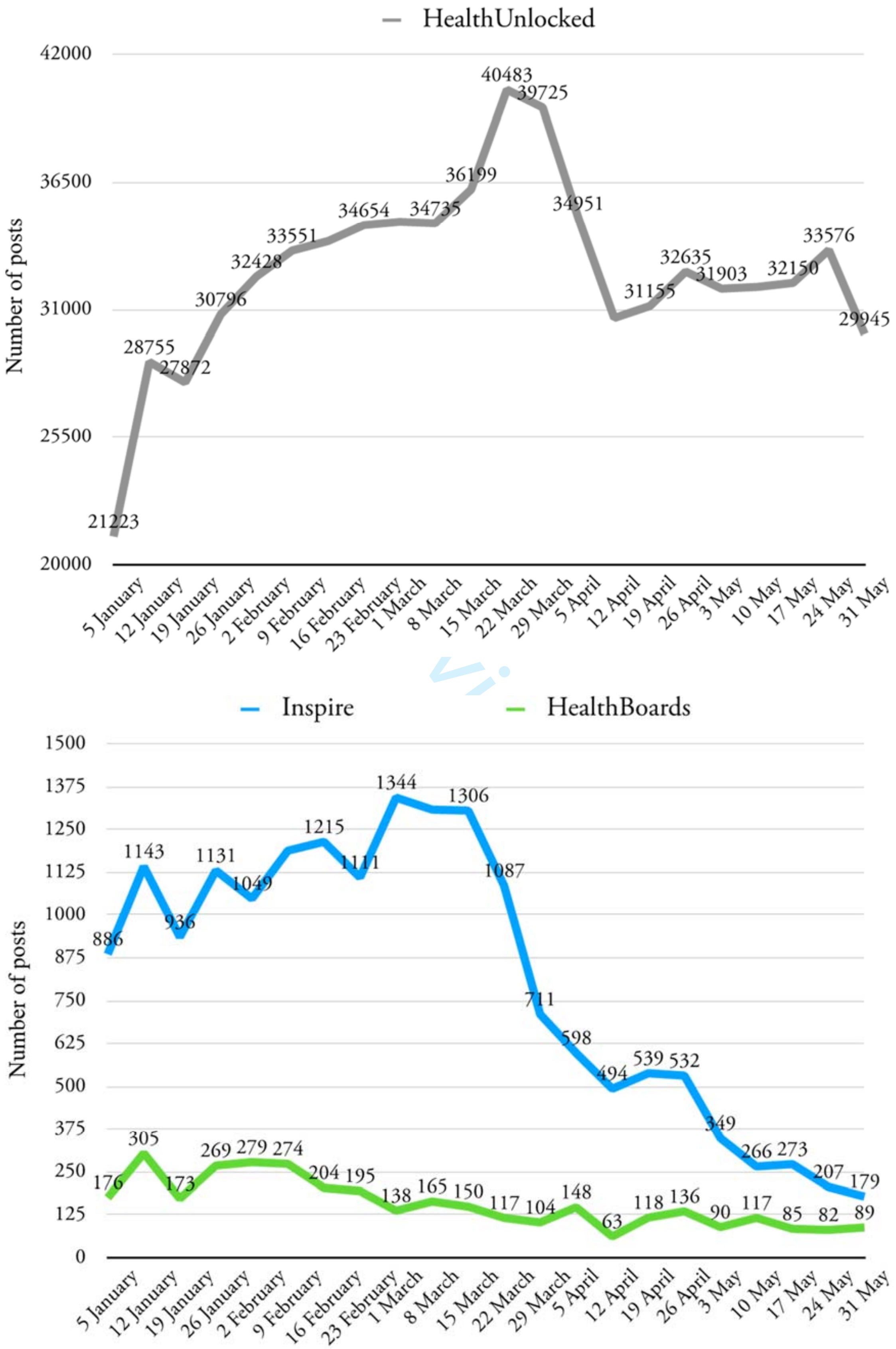

Investigating mental and physical disorders associated with COVID-19 in online health forums: Supplementary Material

Supplementary Figure 2: Percentage of COVID-19 related posts relative to all weekly posts

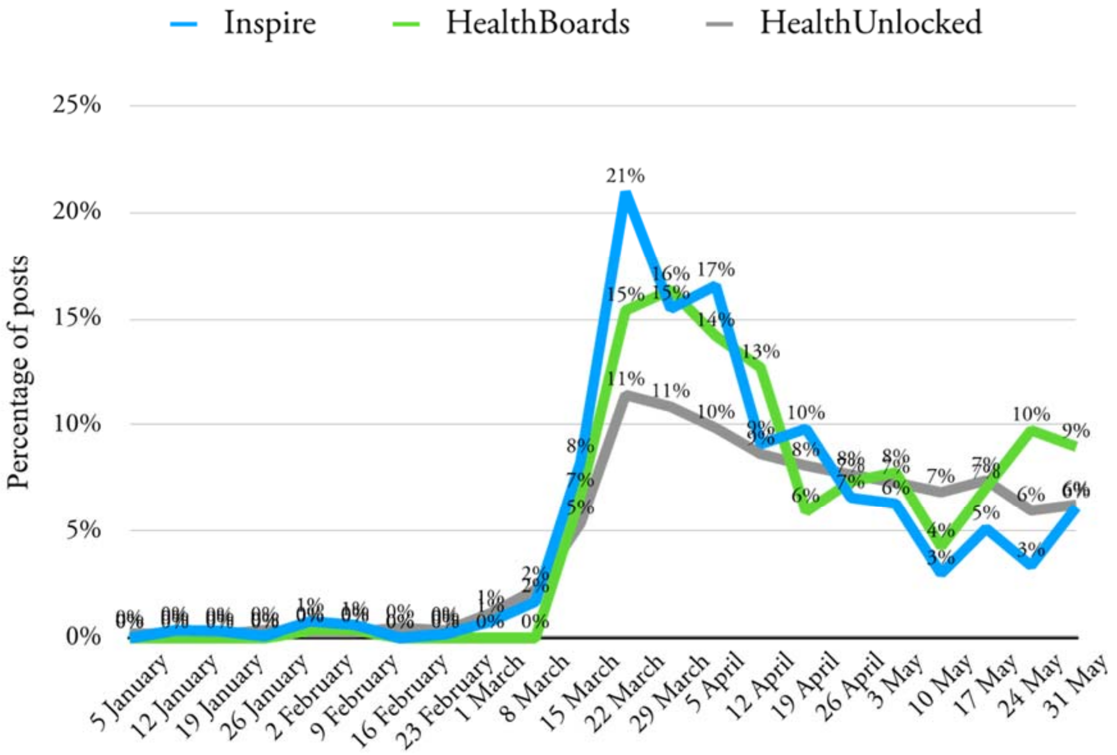

eview only

Investigating mental and physical disorders associated with COVID-19 in online health forums: Supplementary Material

Supplementary Figure 3: Maximum number of COVID-19 related threads active in each given week

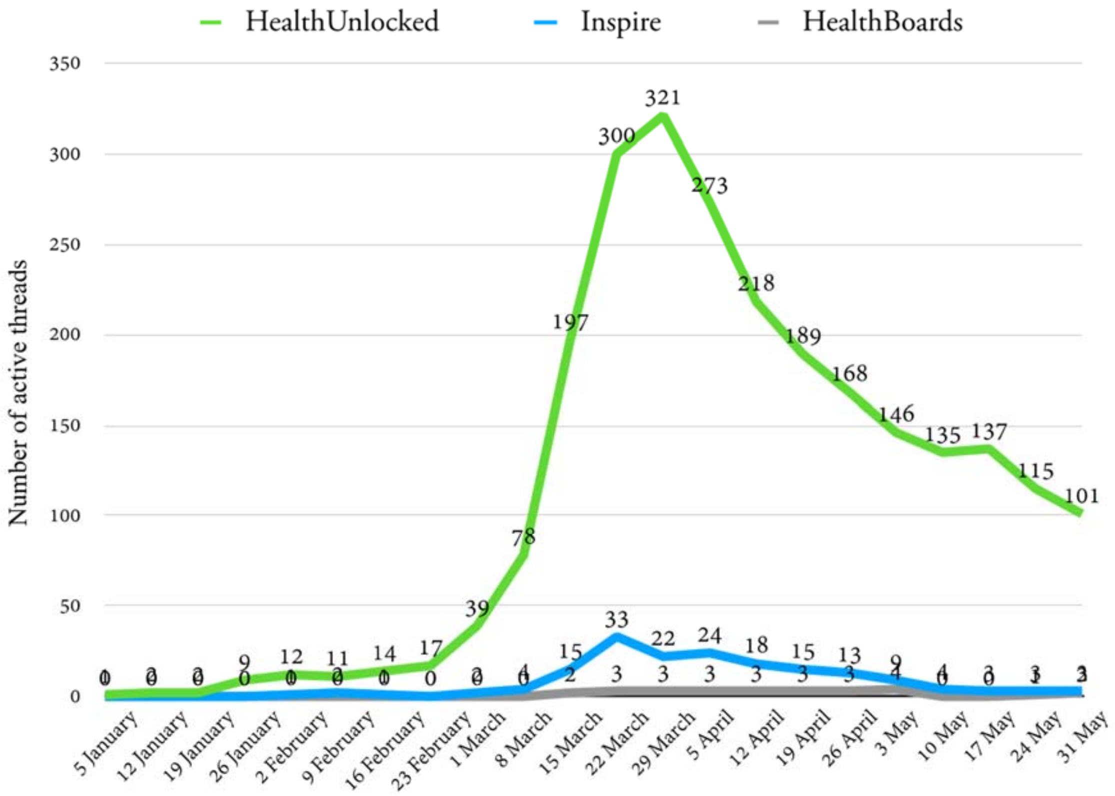

A thread is considered closed and therefore no longer active when there are no further posts from that week within the data collection period (up until 31st May 2020). Note that this does not rule out the possibility of a thread becoming reactivated past this date.

Investigating mental and physical disorders associated with COVID-19 in online health forums: Supplementary Material

Supplementary Figure 4: Proportion of users whose very first post was in a COVID-19 related thread, given weekly

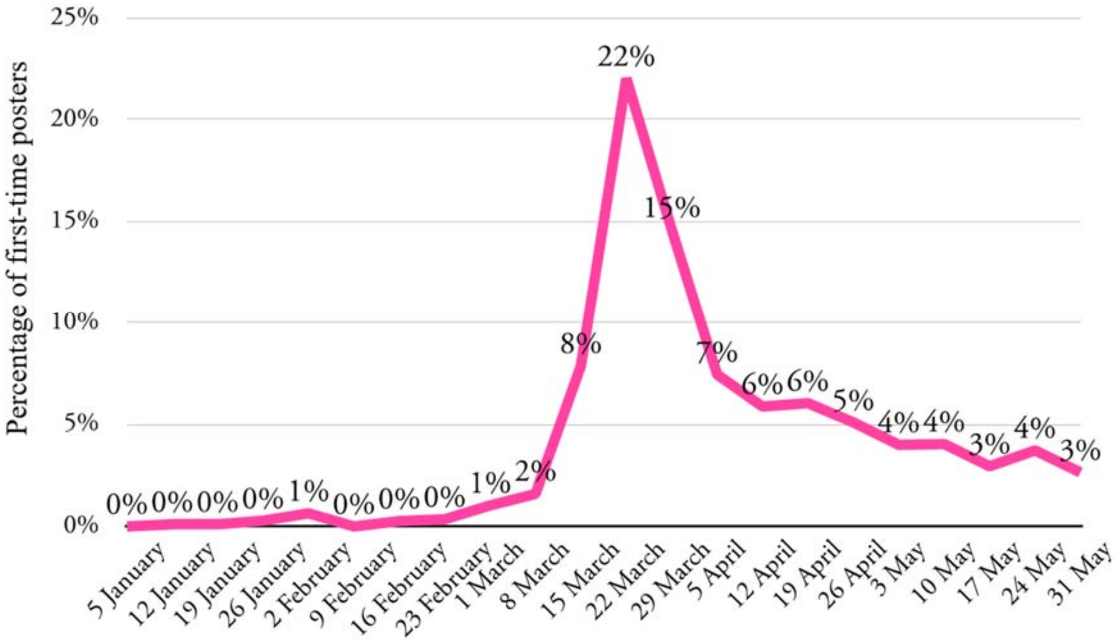

For peer review only
